# Modelling of online shopping behavior in the Czech online environment

**DOI:** 10.1371/journal.pone.0308725

**Published:** 2025-01-15

**Authors:** Veronika Svatošová, Ján Dvorský

**Affiliations:** 1 Faculty of Regional Development and International Studies, Department of Regional and Business Economics, Mendel University in Brno, Brno, Czech Republic; 2 Faculty of Operation and Economics of Transport and Communications, Department of Economics, University of Zilina, Zilina, Slovakia; Al-Ahliyya Amman University, JORDAN

## Abstract

The online environment has its own specifics, which shape the specific behavior of all market subjects, both customers and companies that trade electronically. The aim of the paper is to create, quantify and verify a conceptual comprehensive model of relationships between determinants that influence consumers when shopping online. The impetus for the conducted research was the discovery of the non-existence of a comprehensive model of online shopping behavior that reflects the specifics of the online environment. The main research method is the method of online questioning in the form of a questionnaire survey among a selected group of Czech respondents (n = 926) shopping online with the aim of evaluating the determinants of online shopping behavior. The results of the questionnaire survey are subsequently used to build a comprehensive model of online shopping behavior, which was statistically compiled and verified using the PLS-SEM method, which, based on statistical data, estimates the size and quality of the links between the measured (manifest) and assumed unmeasured (latent) variables. The results show that the selected factors (31 factors) explain up to 82.53% of the variability of the total variance. The results of the correlation analysis of the factors confirmed that the defined factors are not mutually dependent and that in the comprehensive model the factors are not only identified but also statistically significant. The results also confirmed that the correlation of e.g. psychological factors are stronger than dependence on other investigated factors in the comprehensive model of online shopping behavior. The research clearly showed that the key factors for customers when shopping online are Security and risk elimination (SE), together with the Online distribution and logistics (OD) and Online payments (OP). Impulsive online shopping was identified by customers as the least important factor. The validated model provides a comprehensive explanation of the current phenomenon of online shopping that integrates and extends previous studies identifying behavioral models of online shopping behavior.

## Introduction

In 2004, 17% of households used the Internet and 32% of individuals with an Internet connection, or the number increased to 82% in 2020, 83% in 2021 and 85% in 2022. At the same time, this data creates the potential for online sales to grow [1]. The development of the number of Czech individuals who buy regularly or irregularly on the Internet is growing as well [1]. From 3% of users in 2005, their percentage has increased to 54% in 2020 and 61% in 2022, and growth in the number of online shoppers can be predicted in the years to come. The number of users who have never purchased on the Internet also decreased significantly, i.e. by 30% in 2019, compared to 2007, there were 82% of users who had never purchased on the Internet [1]. Since 2019, i.e. the year before the coronavirus pandemic, the number of shoppers has increased by 18% in 2021. According to the E-commerce Report [2], 89% of Czechs have experience with online shopping (in 2022). The EU27 average is also 89%, with people in Denmark having the most online shopping experience (93%) and people with the least experience in Bulgaria (20%). 27% of the Czech Internet population 15+ shop at least once every 14 days and 10% of the Internet population 15+ shop at least once a week [3].

The Czech Republic is one of the fastest growing e-commerce markets in Europe [2], therefore there is great potential to research this phenomenon in this region. In 2020, the Czech Republic experienced a breakthrough year in the field of e-commerce, which used online shopping as a new way for online buyers and sellers during the pandemic. According to the [4], during the coronavirus crisis, 37% of Czech respondents shopped online more, but after the crisis they shop the same as before the crisis. In 2021, the share of these respondents increased to 48% [5]. Furthermore [3], 24% of respondents stated that they have changed their approach to online shopping, they buy more things on the Internet even after the end of the coronavirus pandemic. E-commerce turnover increased by 20% and the number of online merchants increased by up to 8% [5]. Currently, the share of retail sales in e-commerce in total retail sales in the Czech Republic is 16% [3]. The share of Czech e-commerce is expected to increase by 26.4% by 2027 [6]. Czech online customers are now more sensitive to online product discounts earlier than in the past. Changes in consumer behavior due to the rising rate of inflation, the energy crisis and the war in Ukraine are evident from the latest [3] not only in the field of Czech e-commerce, but it can be predicted that this is a temporary change and that a gradual economic stabilization is expected in the coming years, which will also be reflected in online retail sales. In the long term, this crisis in the development of the meaning of e-commerce is unlikely to have a fundamental and long-term effect, especially in terms of changes in online consumer behavior [2].

According to the available information [1–3,6], the results achieved in the area of the Internet community, e-commerce and experience with online shopping on B2C e-commerce markets are comparable to the average for the whole of Europe and the European Union. Czechs are experienced Internet users and online shoppers, and the results achieved for the Czech Republic do not differ from Europe and the European Union, and therefore the results of empirical research conducted on the Czech population can be applied and compared with the average results achieved in Europe and the European Union.

The research gap derives from the following findings of the previous secondary sources: 1) many sources deal separately with the construction and confirmation of behavioral models and models of information technologies, nevertheless few of them integrates constructs (variables) from these both model groups into one integrated model and none of them integrates constructs from these both model groups in the context of online shopping behavior [7–9]; 2) based on previous primary research [10–12], in summary 15 determinants of online shopping behavior have been identified that are served for the designing and verifying the comprehensive model of online shopping behavior as a new innovative construct model in the observed phenomenon; 3) many secondary studies deal with the e-loyalty, however no study integrates this construct to models of online shopping behavior [13–16]; 4) only few studies [17] attempted integrated all latest determinants of online shopping model into one behavioral model or model of information technologies; 5) no study deals thoroughly with modelling of online shopping behavior in the Czech online environment.

The goal of the paper is therefore to fill this research gap in these research aspects and contribute to the further development of the researched issue. The aim of the paper is to create, quantify and verify a conceptual comprehensive model of relationships between determinants that influence consumers when shopping online. This designed comprehensive model of online shopping behavior can help integrate the current diverse range of factors that have previously been studied piecemeal. The main research method is the personal questioning in the form of a questionnaire survey among a selected group of Czech online shopping respondents with the aim of evaluating the development of online shopping behavior over time. The results of the questionnaire survey will subsequently be used to build a comprehensive model of online shopping behavior, which will be statistically compiled and verified using the method of structural equation modeling. The basis for designing the comprehensive model of online shopping behavior is the Davis’s TAM model [18], which is further inspired by the C-TAM-TPB model by Taylor and Todd [19,20] and by the extended Chisnall model [21] by Venkatesh et al. [17]. These models are implemented to the compressive model in its original form including identified constructs and are integrated with identified 15 determinants of online shopping behavior from the previous primary research.

## Theoretical background

This part substantially explain the theoretical background for designing the comprehensive model of online shopping behavior. Shopping behavior models evolve with the development of e-commerce and incorporate individual factors that influence the online shopping process and the final online purchase decision. In the paper, the term abstract or conceptual model is used, which represents a theoretical representation of the system. Conceptual models are often abstractions of phenomena in the real world, whether physical or social [22]. Given the importance of online sales and customers, it is imperative that retailers gain a broad awareness of online shoppers [23]. These models take into account information system factors and the social behavior of online shopping consumers. The basis for the creation of conceptual models determining current online shopping behavior are theories studying the adoption (acceptance) of technology by individuals and the ability to accept new technologies [24].

Entire theories and models of information technology acceptance have been designed to predict user behavior and measure the degree of acceptance and satisfaction of those users with any technology or information system. Predictions and measurements have been made from different perspectives depending on the constructs or variables that present the structure and the areas in which the theories and models have been developed. Therefore, the thirteen theories and models presented here can be divided in two ways: 1) according to the method of their development, 2) according to the scientific field in which they were developed [24]. These two types of classification are made based on the origin of these theories, the history of their development, and the relationship between human behavior and psychology, sociology, and information technology, as shown in the following S1 Table 1 in S1 File. Theories that have developed in the fields of psychology and sociology focus on behavior in technology acceptance, while theories that have been developed in information technology focus on the characteristics of systems and their relationship to technology acceptance [24].

The paper does not define a complete list of all models from the field of social psychology and information technology, but only those that are relevant to the field of e-commerce and online shopping behavior and which serve as inspiration this research, i.e. model TAM, model C-TAM-TPB and extended Chisnall model.

### Model TAM

Information systems have contributed to the existence of a model of technological acceptance (TAM) [18,25], which is an extension of the TRA model [26] and which models how users accept and use technology. Currently, this model is used, for example, to evaluate the success of introducing a new product, software, website, application, etc. Davis’s TAM model [18,25,27] is the most widespread model of technology acceptance and use by users [28]. In the context of online shopping behavior, the TAM model examines the effects of emotional reactions during the first visit to an e-shop with regard to the intention to return later as well as unplanned purchases [24]. The TAM model replaced attitude toward behavior in the TRA model with two measures for technology adoption, namely: perceived usefulness and perceived ease of use that became the basis for the creation of the TAM model. Despite its widespread use, the TAM model has been criticized several times, leading the original proponents to try to redefine it several times.

### Model C-TAM-TPB

The C-TAM-TPB model combines the functionality of the TAM model from the field of information technology and the TPB model [19,20] from the field of social psychology to make better use of the TPB in technology acceptance. This model combines TPB predictors with perceived usefulness from TAM to provide a hybrid model [29]. TAM and TPB theories assumed that behavior is determined by the intention to perform the behavior [18] noted that future research on technology acceptance must address how other variables influence usefulness, ease of use, and acceptance. Taylor and Todd {Citation}assumed that perceived ease of use positively influences perceived usefulness. Both perceived usefulness and perceived ease of use positively influence attitudes [24].

### Extended Chisnall model

Venkatesh et al. [17] defined the current factors that lead to online purchases and formed them into an extended Chisnall model. The goal of the research was again to create a comprehensive model that shapes general and online factors of shopping behavior. The model works with a group of cultural factors (i.e. time availability and time management), demographic factors (age, gender, income, social consumption), economic factors (objective consumption), technological factors (device speed, multimedia capacity, download speed, computer experience, internet experience, online shopping experience) that influence individual psychological factors such as cognition (risk and quality), learning processes (conformity, shopping from home, local shopping), characteristics (price awareness, value awareness, impulse buying behavior), attitude towards shopping (shopping experience, comparison shopping, branded products) and motivation (comfort, enjoyment of browsing online, enjoyment of shopping, shopping for novelties). All these factors lead to intentions to shop online and online shopping behavior. This model evaluates more recent results in relation to factors of online shopping behavior; the model defined a total of fourteen new factors that had not previously been observed in the context of online shopping behavior. The model integrated the results of previous studies that related to the issue of online shopping behavior.

### Determinants of online shopping behavior

However, there is very limited knowledge about consumer behavior on the Internet, as it is a complicated socio-technical phenomenon and includes too many factors [30].

Information and behavioral models that take into account the factors of online shopping behavior are the subject of a number of other researches [9,17,31–33].

Based on the previous research [10], a total of 15 determinants of online shopping behavior were defined, which reflect the current possibilities of online shopping in the Czech online environment (for more details, see S1 Table 2 in S1 File, which, amongst others cites References [34–43]). These determinants are further the subject of this research, which are elaborated into a pre-research own model of online shopping behavior (see S1 Fig 1 in S1 File). The links between the consequences and determinants of online shopping behavior are subsequently formed into a theoretical model of online shopping behavior (see S1 Fig 2 in S1 File), which defines the attitude towards online shopping behavior and subsequently the intention of online shopping behavior and the online shopping behavior. Indirect links of identified determinants of online shopping behavior [15] and online shopping intention and online shopping behavior is determined by the principle of the TAM model.

### Synthesis of model selection into comprehensive model

The following S1 Table 3 in S1 File summarizes the strengths and weaknesses of the selected behavioral models and models of information technologies for designing the comprehensive model of online shopping behavior.

The identified models were selected despite their limitations, which are shown in S1 Table 3 in S1 File. The TAM model has been verified and used in many different studies in the context of online shopping behavior [8,44–46] and used as a basis for further expanding and modified information technology models. The C-TAM-TPB model is a combination of the TPB model from the field of social psychology with the TAM from the field of information technology to achieve a better use of the TPB in technology adoption. It is the first comprehensive model that combines IT and social psychology [24,47] approaches and can be suitably applied to the field of online shopping behavior. The other comprehensive models identified in the S1 Table 1 S1 File (i.e. UTAUT, UTAUT 2) in its original form could not be easily integrated to the conceptual comprehensive models as they are not applicable for online shopping behavior. The extended Chisnall model belongs to the latest comprehensive model explaining current trends in online shopping behavior and respecting external factors of shopping behavior.

Research in this area has resulted in several theoretical models with roots in information systems, psychology, and sociology that commonly explain more than 40 percent of the variance in individual intention to use technology [27,29,48]. Researchers are faced with choosing among a multitude of models and find that they must choose a "favorite model" and largely ignore the contributions of alternative models. Thus, there is a need for revision and synthesis in order to move towards a unified view of user acceptance [7]. The common framework of all behavioral models of information technology are three basic constructs [7]: 1) Individual reaction to using information technology; 2) Intention to use information technology; 3) Actual use of information technology. This common framework is also used for designing the conceptual comprehensive model in this study. An innovative element in the conceptual comprehensive model is the integration and extension of 15 identified and verified determinants of online shopping behavior from the previous primary research into the selected behavioral and informational models. The common framework for all models are the constructs: Attitude towards online shopping, Online shopping intention and Online shopping behavior. Individual constructs and links between them are defined in the theoretical framework of this study. The validated model thus provides a comprehensive explanation of the current online shopping phenomenon that integrates and extends previous studies identifying behavioral models and models of information technologies of online shopping behavior.

## Theoretical framework and research hypotheses

The theoretical framework is based on literature research defining behavioral models that serve as a basis for the creation of models of online shopping behavior and factors of online shopping behavior that created the construct of the comprehensive model of online shopping behavior.

### Variables for creating a comprehensive model of online shopping behavior

The relationships and dependencies in the comprehensive model of online shopping behavior and the identification of research hypotheses that estimate the magnitudes and quality of the relationships between measured (manifest) and hypothesized unmeasured (latent) variables in the comprehensive model of online shopping behavior are described below. The literature review is using the literature primarily focusing on the last decades, nevertheless literature from the period 2000–2010 is also used for the comparison in the observed variables to identify possible changes during the period of e-commerce development.

#### E-security

E-security is determined as a risk that provides a situation, or condition with the potential to indicate economic difficulties based on the modification and misuse of data, damage or non-delivery of products or services, misuse of personal data or any financial loss in online environment [32]. A group of factors dealing e-security were already specified in previous research [10] (S1 Table 2 in S1 File). E-security is closely linked to consumer risk perception. Venkatesh et al. [17] included risk perception in the group of psychological factors, especially the group of perception of agreement (congruence), whereby risk can be perceived as one aspect of e-security. Sheng and Liu [49] showed a direct effect between security and trust in online shopping, that is, the higher the perceived security and risk minimization, the more the trust in online shopping increases, as well as the positive motivation and attitude towards online shopping. Ijaz and Rhee [9] reported that perceived e-risk and e-safety has a positive effect on online shopping intention and attitude. Similar conclusions were also reached by Iglesias-Pradas et al. [50]. Gupta and Dubey [51] add that the basic pillar for creating loyal online customers is ensuring e-security and privacy for both sides of the business transaction. Similar conclusions were reached by. Cui et al. [52] found in their study that higher perceived e-security increases web image perception and e-trust, and these factors have a mediated relationship between e-security and e-loyalty. Eryigit and Fan [14] also confirmed that dimensions of perceived risk have a significant effect one e-loyalty. Based on the above assumptions, the next research hypotheses can be defined: ***H1a*:** *E-security (SRE) is significantly positively affected by the E-loyalty (L)*. ***H1b*:** *E-security (SRE) is significantly positively affected by the attitude toward online shopping (AT)*. ***H1c*:** *E-security (SRE) is significantly positively affected by the E-trust (T)*.

#### E-trust

Trust is generally crucial in many economic activities, which may involve undesirable opportunistic behavior [53]. A group of factors dealing with e-trust were already specified in previous research [10] (S1 Table 2 in S1 File). Geffen et al. [53] use the TAM model to examine the importance of e-trust and find that it is important for the consumer to trust the seller and perceive the store as safe and easy to use, and as trust increases, so does attitude and intention to shop online. Ribadu and Wan ([54] identified write there is a propensity to shop online if people trust of e-shop, website quality, and third-party security. Hasslinger et al. [55] confirmed a positive relationship between trust and online shopping attitude and intention in their model of online shopping behavior. Similar conclusions were reached by Ijaz and Rhee [9]. According to Fernández-Bonilla et al. [56] e-trust is a determinant of e-commerce and online shopping intention and attitude. Several studies have also looked at the relationship between e-trust and e-loyalty. This positive relationship was confirmed by Wilson et al. [16]. Several studies conducted by Nguyen et al., Garepasha et al., Bhat et al. and Wilson et al [15,16,57,58] also found that trust had a positive impact on customer loyalty. Based on these assumptions, the next research hypotheses can be established: ***H2a*:** *E-trust (T) is significantly positively affected by the e-loyalty (L)*. ***H2b*:** *E-trust (T) is significantly positively affected by the attitude toward online shopping (AT)*.

#### E-satisfaction

A group of factors dealing with e-satisfaction were already specified in previous research [10] (S1 Table 2 in S1 File). Sheng and Liu [49] demonstrated in their research the positive direct effect of e-trust and e-loyalty. Furthermore, studies conducted by Sreeram et al. and Wilson et al.[13,16] found that customer satisfaction had a positive impact on loyalty. Selected studies attempted to explain the relationship between user attitudes, satisfaction and behavioral intention to use the system [16,28,48]. In all cases, a positive relationship between the monitored variables was demonstrated. The given arguments create a prerequisite for establishing the next research hypotheses: ***H3a*:** *E-satisfaction (S) is significantly positively affected by the e-loyalty (L)*. ***H3b*:** *E-satisfaction (S) is significantly positively affected by the attitude toward online shopping (L)*.

*General e-factors*. The group of factors dealing with general e-factors were already specified in previous research [10] (S1 Table 2 in S1 File). Hasslinger et al. [55] confirmed in their model of online shopping behavior a positive relationship between the influence of convenience, price and assortment on attitude and intention in online shopping. Similar results were obtained by Bucko et al. (2018), Pilík and Pilík et al. [59–61] where, among other factors, they evaluated that favorable price, convenience and a wide assortment have a positive relationship with the intention and attitude towards online shopping.

Akbar and James and Ijaz and Rhee [9,62], among other factors of online shopping behavior, mentions price, convenience and a wide range of assortment as important factors that influence attitude, intention to shop online as well as overall satisfaction with online shopping. Repeated APEK studies[4,5] came to the conclusion that price, convenience of purchase, time, a wide range of products, possibilities to compare products or services are still one of the key factors that influence the attitude and intention to shop online, secondarily also the overall e-satisfaction. In their studies, Nittala and Agyapong [63,64] found that price and convenience is one of the most important factors for Internet users and creates a prerequisite for repeat purchases on the Internet. Eryigit and Fan [14] confirmed a positive relationship between shopping convenience and e-loyalty, and also confirmed that offering products online has a positive effect on e-loyalty. Based on the aforementioned assumptions, the next research hypotheses can be established: ***H4a*:** *General e-factors (G) is significantly positively affected by the e-loyalty (L)*. ***H4b*:** *General e-factors (G) is significantly positively affected by the attitude toward online shopping (AT)*. ***H4c*:** *General e-factors (G) is significantly positively affected by the e-satisfaction (S)*.

#### Perceived usefulness, perceived ease of use, E-loyalty

The factors perceived usefulness and perceived ease of use were first processed in the TAM model [18,25]. The TAM2 [48], TAM3 [65], C-TAM-TPB [20] and MM [66] models also worked with these factors. These factors are further integrated into the proposed comprehensive model of online shopping behavior. The newly proposed comprehensive model integrates the factor of e-loyalty in connection with perceived usefulness and perceived ease of use, as an important factor of online shopping behavior, which was dealt with by a number of researches [67,68]. Previous researched behavioral models do not integrate the factor of e-loyalty and do not take it into account.

*E-loyalty*, *perceived usefulness*, *perceived ease of use*, *attitude towards use*. Hansen and Jonsson [69] confirmed that there are many factors that create customer loyalty. Studies have identified satisfaction and trust as two important factors in the process of achieving customer loyalty in both the physical and digital worlds [70]. These two variables must be met for the customer to remain loyal to the company.

The research by Wilson et al. [16] demonstrated that perceived usefulness and perceived ease of use had a positive influence on customer satisfaction, trust, and customer loyalty within the Chinese computer industry. In addition, another study conducted by El-Haddadeh et al. [71] also determined a meaningful relationships between ease of use and perceived usefulness for the level of customer loyalty towards specific social networks in China Kim et al. and Fang [72,73] also highlighted the importance and significance of both perceived ease of use and perceived usefulness in influencing and determining consumers’ intentions to use and reuse the same technology and system in the future. Furthermore, In his regression model, Patro [74] also indicated a positive effect of attitude towards online shopping and e-loyalty. Based on these assumptions, the next research hypotheses can also be defined:

***H5:*** *Perceived usefulness (PU) is significantly positively affected by the e-loyalty (L). **H6:** Perceived ease of use (PEU) is significantly positively affected by the e-loyalty (L). **H7:** Attitude toward online shopping (AT) is significantly positively affected by the e-loyalty (L)*.

*Perceived usefulness*, *perceived ease of use*, *attitude towards use*. In Davis’s TAM model, a positive relation between perceived ease of use, perceived usefulness and attitude was demonstrated [18,25]. Perceived ease of use and usefulness affect attitudes toward usability, which shapes intention to use. However, perceived usefulness has a direct effect on intention to use. It is also a fact that behavioral intention influences actual behavior. If a system is not easy to use, it is unlikely to be perceived as useful. Considering this research, the user’s perception of the system’s usefulness and ease of use results in the behavioral intention to use (or not use) the system [18,75]. The TAM model, including its variables, has been tested by many researchers and reported findings agree with this relationship, especially in the context of online shopping behavior modeling [9,32,46,53,76–80].

Based on the stated assumptions, the next research hypotheses can be defined: ***H8*:** *Perceived ease of use (PEU) is significantly positively affected by the perceived usefulness (PU)*. ***H9*:** *Perceived usefulness (PU) is significantly positively affected by the attitude toward online shopping (AT)*. ***H10*:** *Perceived ease of use (PEU) is significantly positively affected by the attitude toward online shopping (AT)*. ***H11*:** *Perceived usefulness (PU) is significantly positively affected by the online shopping intention (OSI)*.

#### Subjective norm

The subjective norm factor was identified by the TRA [26], TPB [81], DTPB [29], C-TPB-TAM [19] and TAM2 [48]. All these models demonstrated a direct positive relationship between subjective norm and online shopping intention. Subjective norm refers to a person’s perception that most people important to him think he should or should not perform a given behavior [26]. The subjective norm has a direct influence on the individual who chooses a certain type of behavior, although he does not perceive this type of behavior as ideal, but with regard to other determinants, he should subjectively behave in this way [48]. The subjective norm is determined by the perceived social pressure from others to behave in a certain way and their motivation to comply with those people’s opinions [82]. According to Taylor and Todd [19], an affection of subjective norm on intentions is anticipated to be stronger for potential users with no prior experience. In contrast, Armitage and Conner [83] criticize the narrow conceptualization of the subjective norms variable, which results in a weak correlation between normative beliefs and intentions. Considering these assumptions, the next research hypothesis can be defined: ***H12*:** *Subjective norm (SN) is significantly positively affected by the online shopping intention (OSI)*.

#### Perceived behavioral control

Perceived behavioral control was processed in the C-TAM-TPB model [19,20], TPB [84], DTPB [29] and RAA [85]. Noar and Zimmerman [86] demonstrated that perceived behavioral control not only influences actual behavior directly, but also influences it indirectly through behavioral intention. According to Taylor and Todd [19], it is expected that online shopping intention (will fully mediate the relationship between perceived behavioral control and online shopping behavior. Ajzen [84] also confirms that intentions are strongly affected by personal factors such as attitudes and perceived behavioral control. The above-mentioned knowledge leads to the establishment of the next research hypothesis: ***H13*:** *Perceived behavioral control (PBC) significantly positively affected by the online shopping intention (OSI)*.

**Attitude toward online shopping.** Attitude toward usability is also indicated as an individual’s positive or negative evaluative affect regarding the performance of a target behavior [87]. In general, behavioral models show that the relationships between attitudes, subjective norms, intentions, and behavior not only predict customer intentions and behavior, but also provide a relatively easy method to find out where and how to focus on changing customer behavior [9]. The relationship between attitude (Attitude toward usability) and behavioral intention was first demonstrated in the TRA model [26]. This relationship has been confirmed many times by other research that dealt with behavioral models, especially in the area of online shopping behavior. Considering the aforementioned assumptions, the next research hypothesis can also be defined: ***H14*:** *Attitude toward online shopping (AT) is significantly positively affected by the online shopping intention (OSI)*.

#### Psychological, cultural, economic and demographic factors

Psychological, cultural, economic and demographic were further defined in Chisnall’s model identifying consumer behavior [21]. Chisnall’s model was further developed by Venkatesh et al. [17], who adapted the model for online consumer shopping behavior and identified the relationship between psychological, demographic, cultural and technological factors on online shopping intention. These factors in connection with the issue of online shopping behavior were also addressed by Hasslinger et al. [55] who demonstrated that online shopping is determined by psychological, cultural, social and personal factors (or a form of demographic factors). The principle of the extended Chisnall model is also used in the construction of a comprehensive model of online shopping behavior. The extended Chisnall model also takes technological factors into account, however, within the proposed comprehensive model, technological factors are included in the group of perceived usefulness and perceived ease of use.

*Psychological factors*. The extended Chisnall model [17] places the greatest emphasis on diverse variable groups of psychological factors that affect the online shopping intention. A number of these variables are included within the other factors of the proposed comprehensive model of online shopping behavior, therefore only selected variables will be selected as part of the psychological factors, namely: congruence, value consciousness, impulsive buying behavior, shopping experience, shopping and browsing enjoyment.

*Congruence*. Due to the fact that the possibilities of purchasing products and services via the Internet are increasing, in this context, the degree of congruence between the Internet as and buying specific product can be defined as a critical factor of online shopping [88]. In the context of online shopping, it can be expected that the congruence between the buyer, the technology (i.e. Internet) and a particular product or service will be decisive in indicating whether online purchase will be the preferred purchase channel for consumers [17].

### Value consciousness

Consumers who are aware of a product’s value are likely to use comparison and exploratory shopping behavior inherent in online shopping to more thoroughly explore product features and price alternatives [89].

### Impulsive buyer behavior

Previous study indicated to the decisive role of impulse buying behavior, i.e. sudden and immediate buying without the intention of purchasing a product or fulfilling a purchase goal [90]. A similar psychological experience can occur when shopping via the Internet for those with higher shopping tendencies [91].

### Shopping experience

The shopping experience plays an important role in facilitating where and when consumers shop and whether and what they buy [92]. Previous studies have found that prior experience is an important determinant of behavior [26,87]. A previous study [9] evaluated that online shopping experience could only be gained by customers through previous purchases, and online purchases strongly depended on past online shopping experience, whereby shoppers decided whether to purchase from an online retailer they had previously they shopped.

### Shopping and browsing enjoyment

Online purchase can provide a great potential of personal pleasure [93]. A previous study also showed that shopping enjoyment positively affects the intention to return to the website [94] and purchase intentions [95].

Therefore from above all the hypothesis can be defined: ***H15*:** *Psychological factors (PF) is significantly positively affected by the online shopping intention (OSI)*.

#### Cultural factors

The literature review found that although family or ethnic culture may influence psychological purchasing factors. The extended Chisnall model [17], on important lifestyle attributes influencing psychological factors, namely time availability and time management [17]. Internet users who have an active life and little free time are likely to choose an appropriate shopping channel, as the possibility of the Internet’s limitless possibilities in terms of place and time may induce those who suffer from a lack of time to considering online shopping [17]. Therefore, the next hypothesis is: ***H16*:** *Cultural factors (CF) is significantly positively affected by the psychological factors (PF)*.

#### Economic factors

Consumers buy a good if the value provided by the product or service equals or exceeds the economic investment in the good. Economic factors includes objective consumption based on the model of Venkatesh at al. [17] and net disposable income that complement this category [55,79,90].

### Objective consumption

Objective consumption provides the significance a consumer attaches to functional and economic issues before purchasing products [17]. As a result, consumers that evaluate objective consumption are probably retained in exploratory and comparison shopping, leading to a wider choice and knowledge of available goods and services [17].

### Disposable income of Internet users

Online buyers with higher disposable income are more retained in impulse purchases and shop more often at home [90]. Online buyers with higher disposable income are also more likely to engage in online purchasing and spend more money per purchase [17,79]. Online shoppers with higher household incomes have tendency to buy more online compared to consumers with lower incomes. This is because higher household incomes are often positively affected with computer ownership, Internet access and higher levels of consumer education [76]. Therefore, the next hypothesis is: ***H17*:** *Economic factor (EF) is significantly positively affected by the psychological factors (PF)*.

#### Demographic factors

Psychological factors associated with shopping can be influenced by a number of demographic factors, among which the social class structure of the consumer, the life cycle and the influence of the family or reference group of the group can be prioritized. The group of demographic factors was also inspired by extended Chisnall model [17] taking into account mainly the age, gender, disposable income and education of Internet users.

### Age of internet users

Age id determined as a crucial factor in online purchasing preferences and perceptions [96]. Older customers have tendency to by more price conscious [17]. Internet facilitates access to products and services that are inconvenient to buy [59], and older buyer tend to be more stressed than younger consumers [17,97]. Younger adults (mainly under 25 years old), are keen on using new technologies such as the Internet to learn about new goods and services, searching product information, compare and evaluate products alternatives [76].

### Gender of internet users

The gender of users is traditionally one of the most closely monitored demographic factors in various scientific fields. Women are processing complex information, while men select more [98].

### Education

Consumers with higher education are more comfortable shopping online [99]. Higher education has also been shown to lead to higher disposable income, which is positively correlated with Internet access and computer ownership [76]. Other studies have shown there is a significant positive correlation between a higher level of consumer education and increased interest in online purchasing [100,101] found that consumers who have a higher level of education are more likely to shop online than those who have a lower level of education.

Considering the above-mentioned characteristics, the next main research hypothesis can be defined: ***H18*:** *Demographic factors (DF) is significantly positively affected by the psychological factors (PF)*.

#### Online shopping intention

Previous studies indicated that intention have an important position in predicting behavior [17,65,102], and this direct relationship has been demonstrated in many behavioral models [17,18,26]. Direct experience leads to a more significant and more steady relation between intention and behavior [26]. Based on these assumptions, a hypothesis can be formulated: ***H19*:** *Online shopping intention (OSI) is significantly positively affected by the online shopping behavior (OSB)*.

#### Proposal of a comprehensive model of online shopping behavior

The identified determinants of online shopping behavior, which were already presented in detail in the previous chapter, enter the comprehensive model (15 in total, see S1 Fig 1 in S1 File) together with variables from theoretical framework. The psychological, demographic, economic and cultural factors were taken from Chisnall’s extended model [8], reduced and transformed for the needs of this comprehensive model (see S1 Fig 2 in S1 File).

## Materials and methods

The aim of the article is to create, quantify and verify a comprehensive model of relationships between determinants that influence the consumer when shopping online.

### Data collection

The main goal of the selected method is to evaluate the attitudes towards individual factors that influence the respondent when shopping online. The questionnaire could be completed by a Czech consumer over the age of 15 who had made at least one online purchase in the last 12 months (hereinafter referred to as the "respondent").

A questionnaire survey can address a representative sample of respondents, or a smaller part of a group that is typical of the entire population. The results obtained from a representative sample of respondents can then be generalized for the entire surveyed population. Questionnaires often have standardized answers, do not require as much effort on the part of the interviewer as oral or telephone surveys, and often have standardized answers that facilitate data compilation. The main advantage of a questionnaire survey is the relatively low time and financial requirement. The results of a questionnaire survey can be representative for the rest of the population and can be processed statistically. A disadvantage of questionnaire survey can be the intentional or unconscious risk of distortion of perception on the part of respondents, who communicate only their individual view of the monitored situation [103]. This research counts on this limitation of quantitative research with comparison of answers of the similar research and the validation of findings were discussed and confirmed by experts of Association for Electronic Commerce and Smart Network that closely cooperated on collection of data.

The creation and implementation of the questionnaire was carried out in the next phases. The preparatory phase took place during the 7rd and 12th months of 2022. Its content was a detailed processing of the literature search of current case studies from primary and secondary sources devoted to the given issue. The methodological phase focused on defining: i. the purpose of the questionnaire; ii. research hypotheses; iii. factors and their claims (duration: 12 months– 7/2022–7/2023). The pilot research was carried out during three weeks (from 15/1/2023 to 05/2/2023) in order to verify the relevance, comprehensibility and formulation of the statements in the questionnaire. The questionnaire was distributed to respondents by e-mail and through social networks (Facebook, LinkedIn). It was attended by 25 respondents (14 women and 11 men). The selected statements were reformulated based on the respondents’ suggestions.

The data collection itself took place from 19/02/2023 until 09/05/2023. In the first phase, 328 questionnaires were collected with an average return rate of 9.5% (3,452 respondents were contacted). Due to the fact that a sufficient number of respondents was not obtained, the next phase of data collection was implemented. In this phase, the respondents were contacted by phone with a request to fill in the questionnaire. (return rate 9.5%; n = 328). 354 respondents participated in the second phase. The cumulative rate of return (first phase + second phase) was 19.75%. Considering the improvement of the representativeness of the sample set (or the structure of the sample set of respondents corresponded with the structure of respondents in the Czech Republic), the third phase of data collection was implemented. Using the method of reference selection, 244 questionnaires were filled out through publication on social networks of associations (e.g. Smart Network, APEK, etc.). According to Raosoft [104], the representativeness of the research sample with 385 respondents is ensured at a confidence interval of 95% and a permissible statistical error of 5%. However, in order to ensure the validity, composability and statistical verification of the comprehensive online shopping behavior model (KMON), it is recommended to ensure at least double the number of respondents, i.e. 770 respondents, so that the method of structural equation modeling (SEM) can be used. Non-proportional quota selection therefore assumes securing a minimum representative sample of 385 respondents, ideally securing 770 respondents. The representativeness of data is thus ensured (n = 926).

#### Questionnaire and variables

The questionnaire contained 109 questions. Four socio-demographic questions were formulated at the beginning of the questionnaire. Namely gender (male, female), age (15 to 24 years; 25 to 39 years; 40 to 54 years; 55–76 years; 77 and over); education (primary; high school without a high school diploma; high school with high school graduation; university ‐ I. and II degrees; Ph.D., Dr. and similar) and net monthly income (up to 625 euros; 626–1,250 euros; 1,251–1,875 euros); 1876–2,500 euros; 2,501 and more euros) of the respondent. This demographic division of respondents is given according the division of Czech Statistical Office. According to APEK [3], the age group of 25 to 34 buys most often online, the age group of 15 to 24 years ranked second. Age group 15–18 years, or 15–24 years old is also included in the research and is relevant for the research. We are based on CSO and APEK data [1,3], which also regularly include respondents older than 15 years in the research.

The questionnaire survey took place in three phases. In the first two phases, the proportional quota selection method according to the age group of respondents was used. In proportional quota sampling, the main characteristics of the population are represented by sampling them with respect to their share in the population under study [105]. The selection of respondents took place in the third phase of the questionnaire survey using the reference sampling method, which uses the participants’ social networks to access specific populations [106].

The next part of the questionnaire contained 105 statements that refer to 31 factors that are believed to influence the respondent when shopping online. The respondent had to answer each statement with one of the next options (according to Likert scale ‐ 5 points): 1 ‐ I completely disagree, 2 ‐ I rather disagree, 3 ‐ I neither agree nor disagree, 4 ‐ I rather agree, 5 ‐ I completely agree. All questions and statements in the questionnaire were marked as mandatory and all were closed with the possibility of choosing answers. Statements that evaluate attitudes and factors towards online shopping behavior were purposefully asked with regard to the identification of factors that serve as a starting point for the creation of a comprehensive model of online shopping behavior. Three to five statements were asked for each factor, which can comprehensively assess the respondent’s attitude to the given situation. These claims have been inspired by a number of previous researches dealing with information systems modeling [7,8,18–20,24–26,29,48,81] and behavioral models focusing on online shopping behavior [9,32,80,107].

The next factors were investigated (number of statements; see appendix): attitude toward online shopping (AT, 3); online shopping intention (OSI, 4); online shopping behavior (OSB, 5); perceived usefulness (PU, 4); perceived ease of use (PEU; 4); subjective norm (SN; 3); perceived behavioral control (PBC; 3); e-loyalty (L; 3); e-security: i. security and risk elimination (SRE; 4); ii. online payments (OP; 3); iii. online distribution and logistics (OD; 3); e-satisfaction: i. websites and web design (WW; 5); ii. online communication (OC; 3); iii. online visualization and product description (OV; 5); iv. customer service (CS; 3); in. multichannel sales (MC; 3); general e-factors: i. lower prices (LP; 3); ii. unlimited time and convenience of purchase (UT; 4); iii. wider assortment offer (WAO; 3); e-trust: i. trust in e-shop (TE; 4); ii. e-shop certification (EC; 3); iii. product references (RP; 3); iv. e-shop references (ER; 3); psychological factors: i. congruence (C; 3); ii. value consciousness (VC; 3); iii. impulsive buyer behavior (IBB; 3); iv. shopping experience (SE; 3); in. shopping and browsing enjoyment (SBE; 4); cultural factors: i. time availability (TA; 3); ii. time management (TM; 3): economic factors: i. objective consumption (OCO; 4) and ii. net monthly income (NDI) and demographic factors: i. gender; ii. education; iii. age.

#### Structure of respondents

The structure of the respondents (n = 926) according to socio-demographic characteristics is as follows: gender of the respondent– 428 (46.2%) men, 498 (53.8%) women; respondent’s education– 95 (10.3%) basic education; 228 (24.6%) secondary education without high school diploma; 283 (30.6%) secondary school education with high school diploma; 320 (34.5%) higher university education–Ph.D., Dr. and so on); age of the respondent: 264 (28.5%)–age from 15 to 24 years; 246 (26.6%) age from 25 to 39 years; 229 (24.7%) age from 40 to 54 years; 124 (13.4%) age from 55 to 76 years; 63 (6.8%) over 77 years; net monthly income– 154 (16.6%) with a net income of up to 625 euros; 243 (26.3%) with net income from 626 to 1,250 euros; 263 (28.4%) with net income from 1,251 to 1,875 euros; 165 (17.8%) with a net income from 1876 to 2,500 euros; 101 (10.9%) with a net income of more than 2501 euros.

S2 Table 4 in S1 File shows the percentage distribution of individual age groups according to the CSO [1] and the proportional minimum required number of respondents in each age group to achieve representativeness of the monitored sample (n = 385) and the ideal required number of respondents in each age group (n = 770). The table also contains the actual number of respondents obtained in all age groups (n = 926) for all three phases of the questionnaire survey. According to the CSO [1], the Czech Republic had a total of 10,516,707 inhabitants in 2022, of which 83.9% are residents over the age of 15 (i.e. 8,823,299 inhabitants of the Czech Republic over the age of 15). If it can be assumed that 89% of Czech respondents over the age of 15 have experience with online shopping in the last year, as found by the APEK and CSO [1,4], then the basic research set is 7,852,736 respondents in the Czech Republic. The percentage distribution of potential respondents by generational cohorts is shown in S2 Table 4 in S1 File.

#### Statistical methods

The Partial Least Squares Structural Equation Modeling (PLS-SEM) method is applied to evaluate and verify the formulated hypotheses (see conceptual model of consumer purchasing behavior). PLS-SEM is currently used in many scientific researches and case studies, e.g. [9,17,55,108]; which deal with the issue of online shopping behavior of customers.

In the first step, the descriptive characteristics of all factors were calculated in order to get an initial idea of the sample set and to verify the multiple normal distribution. Descriptive characteristics such as mean, standard deviation, skewness, and kurtosis were calculated. If the values of skewness and kurtosis are in the interval of values from -2 to 2, then there is a strong assumption that the variable comes from a normal distribution (see S1 Table 2 in S1 File).

In the second step, a multivariate method such as factor analysis (CFA, EFA) was applied to determine the links and connections between statements and factors (or classification of factors). To prove the consistency, validity and reliability of the selected statements to the defined factors, the next were applied (according to Hair et al., 2006): Cronbach’s Alpha (CA; minimum value (MV) = 0.7); Composite reliability (CR; MV = 0.7); Average variance extracted (AVE; MV = 0.5); Corrected item–Total correlation (CI-TC; MV = 0.5); Factor loading (FL; MV = 0.5); Keiser-Meyer-Olkin Measure of sampling adequacy (KMO test; MV = 0.5) and Bartlett’s test of sphericity (BTS; MV < significance level). The method of Principal component analysis with Varimax rotation (and maximum 25 iterations; [108]) was used for factor extraction. The decision on the number of factors was determined with the help of a scree graph [109] and BIC–Schwarz’s Bayesian information criterion) [110].

The PLS-SEM method is suitable for evaluating hypotheses or relationships (see S1 Fig 1 in S1 File) in a comprehensive model of online shopping behavior. A comprehensive model is a combination of regression and factor analysis, which results in latent factor regression [111] In the comprehensive model of online shopping behavior, it is possible to identify and quantify comprehensive not only direct, but also indirect relationships between latent variables and between latent and manifest variables [112]. All necessary tests and calculations were carried out in the program IBM SPSS Statistics vs. 28 and IBM SPSS Amos.

## Results

The results of selected descriptive characteristics (M–Mean; SD–Standard Deviation; SKE–Skewness; KUR–Kurtosis) of all investigated variables (I–items) are summarized in the S3 Table 5 in S1 File. The results (see S3 Table 5 in S1 File) show that customers agree the most with the statement that "they shop online mainly because it allows me to buy specific products/services" (M = 4,551). On the contrary, the biggest disagreement is with the statement that "my cupboards are full of unused products that I bought online" (M = 1,590). The greatest variety of customer responses is to the question "How many products do you usually buy per online purchase?" (SD = 1.563). The five most positively evaluated factors according to customers include (descending by M): online visualization and product description (M = 4,270); online distribution and logistics (M = 4,236); customer service (M = 4.189); security and risk elimination (M = 4.141); online payments (M = 4.121). The five most negatively evaluated factors according to customers include (in ascending order according to M): impulsive buyer behavior (M = 1.744); time management (M = 2,230); multichannel sales (M = 2,911); online shopping behavior (M = 3.014); lower prices (M = 3.014). The five most consistently evaluated factors according to customers include (in ascending order according to SD): value consciousness (SD = 0.422); online communication (SD = 0.509); impulsive buyer behavior (SD = 0.511); congruence (SD = 0.534); time availability (SD = 0.562). The five least consistently evaluated factors according to customers include (descending by SD): attitude toward online shopping (SD = 1.222); online shopping behavior (SD = 1.066); unlimited time and convenience of purchase (SD = 1.009); perceived ease of use (SD = 1.007); online communication (SD = 0.979).

The results (see S3 Table 5 in S1 File) confirmed that all investigated variables fulfilled the assumption of the probability model of normal distribution, which is a prerequisite for a deeper statistical evaluation using the SEM methodology.

Results of verification of validity and reliability of factors and statements in the questionnaire using CA ‐ Cronbach’s Alpha (critical value ‐ CV; CV > 0.7); CR–Composite Reliability (CV > 0.7); AVE–Average Variance Extracted (CV > 0.5); CI-TC–Corrected Item-Total Correlation (CV > 0.5); FL–Factor loading (CV > 0.5); C–Communality (CV > 0.5); KMO–Kaiser-Meyer-Olkin test (CV > 0.5) are the subject of S3 Table 6 in S1 File.

The results (see S3 Table 6 in S1 File) show that the internal consistency between the statements and the defined factors is acceptable for the next factors: AT; OP; OC; L; FROM; CS; MC; WAO; PR; C; LP; EC; ER; TM; AXIS; PEU; UT; PU; WED; OC; OSB; WW and OV. After excluding PBC3 (CI-TC = 0.376; C = 0.272) from the PBC factor, satisfactory results were obtained for the factor (PBC): CA = 0.882; CI-TC = 0.789; C = 0.894; FL = 0.946; KMO = 0.500. After removing SN3 (CI-TC = 0.376; C = 0.375) from factor SN, satisfactory results of factor (SN) were obtained: CA = 0.925; CI-TC = 0.860; C = 0.930; FL = 0.964; KMO = 0.500. After removing VC1 (CI-TC = 0.203; C = 0.180) from the VC factor, satisfactory results of the factor (VC) were obtained: CA = 0.814; CI-TC = 0.690; C = 0.845; FL = 0.919; KMO = 0.500. After removing SE1 (CI-TC = 0.460; C = 0.455) from the SE factor, satisfactory results of the factor (SE) were obtained: CA = 0.993; CI-TC = 0.989; C = 0.993; FL = 0.996; KMO = 0.500. After removing IBB3 (CI-TC = 0.384; C = 0.449) from factor IBB, satisfactory results of factor (IBB) were obtained: CA = 0.780; CI-TC = 0.640; C = 0.820; FL = 0.906; KMO = 0.500. After removing TA1 (CI-TC = 0.117) from the TA factor, the best results of the validity and reliability of the factor (TA) were achieved: CA = 0.725; CI-TC = 0.578; C = 0.789; FL = 0.888; KMO = 0.500. After removing TE2 (CI-TC = 0.360; C = 0.393) from the TE factor, satisfactory results of the factor (TE) were obtained: CA = 0.822; TE1: CI-TC = 0.989; TE3: CI-TC = 0.534; TE4: CI-TC = 0.734; TE1: C = 0.855; TE3: C = 0.573; TE4: C = 0.791; TE1: FL = 0.925; TE3: FL = 0.757; TE4: FL = 0.890; KMO = 0.637. The analysis of the SBE factor (SBE1, …, SBE4) results in the next findings: statements SBE1 (CI-TC = 0.408) and SBE 2 (CI-TC = 0.114) do not form the mentioned factor. The SBE factor is formed by statements SBE3 and SBE4 with the next results: CA = 0.936; CI-TC = 0.879; C = 0.940; FL = 0.969; KMO = 0.500.

The results of the KMO test and Bartlett’s sphericity test without the above-mentioned indicators are the subject of S4 Table 7 in S1 File. The results of the KMO test (see S4 Table 7 in S1 File) confirmed that a proportion of the variance of individual variables (items) can be explained by background factors. This is because the value of the KMO test (KMO = 0.870) is close to the value of 1, or a large proportion of the variance is explained by the factors. Also, the results of Bartlett’s test of sphericity (p-value = 0.000) are accepted at the level of significance (α = 1%).

The results of the explanation of the total variance of the factors (and their items) is the subject of S4 Table 8 in S1 File. Results from S4 Table 8 in S1 File show that the selected factors (31 factors) explain up to 82.53% of the variability of the total variance. The remaining 17.47% of the variability of the total variance can be explained by indicators and factors that are not in the S4 Table 8 in S1 File. Based on the results (S4 Tables 8 and S5 Table 9 in S1 File), all factors were proven and identified. These results are also supported by a scree graph (see S4 Fig 3 in S1 File), which also confirmed the stated number of factors (using Kaiser’s rule ‐ more than 1% of the total variance; Bentler, 1990).

The results of the factor correlation analysis confirmed that the defined factors are not interdependent (VIF values) and that in the comprehensive model the factors are not only identified but also statistically significant. The results also confirmed that the correlation of e.g. psychological factors (C, VC, IBB, SE, SBE) is stronger than the dependence on other investigated factors in the comprehensive model of online shopping behavior. Analogously, this also applies when evaluating other factors that are linked to the main factor, e.g. (socio-demographic factors, or e-trust). Due to robustness, the discriminant validity correlation matrix is not included in the tables.

The results of discriminant validity analysis with applying Fornell-Lacker criterion confirmed that diagonal values (the square root of the variance shared between the factors and their AVE measures) are larger in comparison with off-diagonal values (correlation between factors). Now we proceed to verify the statistical significance of the formulated hypotheses (H–hypothesis, Coef.–coefficient; Sig.–significance, SE–Standard error; CR–Critical ratio), which is the subject of S3 Table 6 in S1 File and the evaluation of the formulated hypotheses (S5 Table 9 in S1 File). If the p-value of the t-test (critical ratio) is lower than the significance level of 5% (the most frequently used significance level in economic sciences), then there is a statistically significant influence (>>; see S3 Table 6 in S1 File) of the independent variable on the dependent or we accept hypothesis (H) (see S3 Table 6 in S1 File). If the p-value of the t-test is higher than the significance level of 5%, then we reject the hypothesis (H) (see S5 Table 9 in S1 File). A positive value of the regression coefficient (path coefficient) indicates a positive influence of the independent variable on the dependent variable. The final model (see S5 Fig 4 in S1 File) provides its hypotheses verification based on structural equation modelling.

## Discussion

The research focused on building and verifying a comprehensive model of online shopping behavior, based on which 19 statistical hypotheses were defined. **Hypothesis H1a** did not confirm the positive effect of e-security on e-loyalty. Previous studies have repeatedly confirmed the direct relationship between e-security and e-trust [52]. Other studies, on the other hand, have shown a positive relationship between e-security and e-loyalty mediated through e-trust [51,52]. Eryigit and Fan [14] demonstrated a direct relationship between e-security and e-loyalty. However, this research did not confirm these assumptions (**hypothesis H1b, hypothesis H2a**).

**Hypothesis H2b** was clearly confirmed, that is, e-security has a positive effect on attitude toward shopping behavior, i.e., the higher the perceived e-security, the higher and better the attitude toward online shopping. These conclusions are also accepted by Ijaz and Rhee (2018), who state that perceived risk and e-security have a positive effect on online shopping intention and attitude. Similar conclusions were also reached by Iglesias-Pradas et al. [50]. It can therefore be evaluated that e-security is a decisive factor that positively affects the attitude towards online shopping.

**Hypothesis H1c**, that e-security has a positive effect on e-trust, was only partially confirmed (economic perspective). From a statistical point of view, this hypothesis was not confirmed. This hypothesis consists of 12 sub-hypotheses. Within the framework of partial hypotheses, only a positive effect of security and risk elimination on trust in e-commerce (e-shop) was proven, i.e., if the online shopper perceives the risks associated with online shopping as low, then trust in e-commerce and probably in the entire e-commerce increases trading, as well as the positive influence of online distribution and logistics on e-shop certification. Ho and Oh (2008) confirmed that e-shop certification increases trust in online shopping, which partially coincides with the conclusions of this research.

The other sub-hypotheses were not confirmed, and therefore the H1c hypothesis cannot be unequivocally confirmed, but neither can it be refuted. Sheng and Liu [49], who showed a direct effect between safety and trust in online shopping, that is, the higher the perceived security and risk minimization, the more the trust in online shopping increases. The same conclusions were reached by Ijaz and Rhee [9], who also confirmed a positive relationship between e-security and e-trust in their model of online shopping behavior. Similar conclusions were also reached by Iglesias-Pradas et al. [50]. Ribadu and Wan [54] confirmed that there is a propensity to shop online if people trust the online store, website quality, and third-party security. Several studies have also looked at the relationship between e-trust and e-loyalty. This positive relationship was confirmed by Wilson et al. [16].

Several studies conducted by Nguyen et al. (2013), Garepasha et al. and Bhat et al. and Wilson et al [15,16,57,58] also found that trust had a positive impact on customer loyalty. Based on these studies, it was hypothesized that e-trust positively affects e-loyalty. However, this research surprisingly did not confirm this positive relationship (**hypothesis H2a**). Research has confirmed a positive relationship between e-security and e-trust (**hypothesis H1c**), and further between e-satisfaction and e-loyalty (**hypothesis H3a**).

The **hypothesis H2a** that e-trust positively influences online shopping attitude was partially confirmed. This hypothesis consisted of four sub-hypotheses, three of which were confirmed. On the contrary, e-shop certification, product references and e-shop references clearly have a positive relationship with attitudes towards online shopping. According to Fernández et al. [56] e-trust is a determinant of e-commerce and online shopping intention and attitude towards online shopping. Ribadu and Wan [54] write about the importance of e-trust, i.e. there is a propensity to buy online if people trust the online store, website quality and third-party security. These conclusions are also confirmed by previous studies, which rather focused on general trust in e-commerce and its relationship to online shopping attitude [53–56].

Research has clearly confirmed a positive relationship between e-satisfaction and e-loyalty (**hypothesis H3a**). The research confirmed that all the mentioned factors based on literature research are an important prerequisite for the emergence of e-satisfaction. These factors of e-satisfaction create a positive prerequisite for the emergence of e-loyalty. A number of previous researches have again confirmed the positive relationship between e-satisfaction and e-loyalty [13,16], as has this research.

Another clear positive relationship was also demonstrated between e-satisfaction and attitudes towards online shopping (**hypothesis H3b**). Also, previous studies have explained the relationship between user attitudes, satisfaction and behavioral intention to use the system [16,28,28,53].

The research clearly confirmed a positive relationship between general e-factors and e-loyalty (**hypothesis H4a**). Nittala [63] and Agyapong [64] found that price is one of the most important factors in creating e-loyalty. Agyapong [64]states that convenience is a factor that leads to e-loyalty. In their research, Eryigit and Fan [14] confirmed a positive relationship between shopping convenience and e-loyalty, and also confirmed that offering products online has a positive effect on e-loyalty.

The research also confirmed a definite positive relationship between selected general e-factors and attitudes towards online shopping (**hypothesis H4b**). Similar results were obtained by Bucko et al. Pilík, Pilík et al. Chaffey et al., Akbar et al. and Ijaz and Rhee [9,59–62,113], among other factors of online shopping behavior, mention price, convenience and a wide range of products as important factors that influence attitude, intention towards online shopping. The conclusions of previous studies confirm the results of this research as well.

However, the research did not confirm a clear positive relationship between selected general e-factors and e-satisfaction (**hypothesis H4c**). This hypothesis consists of 15 sub-hypotheses that address the interrelationship between general e-factors and e-satisfaction factors. 6 sub-hypotheses were confirmed of these 15 sub-hypotheses. A positive relationship between a wider assortment product and the perception of web design and orientation on websites was clearly demonstrated. Furthermore, a positive relationship was demonstrated between the unlimited time of purchase and the possibility of interactive online communication, as well as between a wider range of products and the possibility of interactive online communication. A positive relationship was also demonstrated between lower prices and online product visualization, then a wider assortment of products and online product visualization, and finally between lower prices and added value to the purchase (customer service). The results of previous studies between general e-factors and e-satisfaction are also not clear-cut and mostly dealt with this relationship marginally, and these studies mostly found a mediated or indirect relationship of selected factors to e-satisfaction. For example, repeated APEK studies [3,5] came to the conclusion that price, convenience of purchase, time, a wide assortment of products, possibilities to compare products or services are still one of the key factors that influence the attitude and intention to online shopping, secondarily also overall satisfaction with online shopping. Nittala [63] and Agyapong [64] found in their studies that price is one of the most important factors for Internet users and creates a prerequisite for repeat purchases on the Internet and creates an indirect positive relationship with e-satisfaction.

The factors (**hypothesis H5,H6, H7**) perceived usefulness and ease of use are based on the TAM model [18,25], which were subsequently elaborated in other information and behavioral models [20,29,48,65,66]. In the proposed comprehensive model, e-loyalty was also incorporated. However, no research integrates e-loyalty into informational or behavioral models. However, some research addresses the general relationship between perceived usefulness, ease of use, and e-loyalty in general information systems models. For example, research by Wilson et al. [16] indicated that perceived usefulness and perceived ease of use had a positive and meaningful influence on customer satisfaction, trust, and customer loyalty. Furthermore, for example, El-Haddadeh et al. [71] indicated meaningful relation found significant relationships between ease of use and perceived usefulness for the level of customer loyalty. Kim et al. [72] and Fang [73] also highlighted the importance of both perceived ease of use and perceived usefulness in influencing and determining consumers’ intentions to use and reuse the same technology and system in the future. In his regression model, Patro [74] also indicted a positive influence of attitude towards online shopping and e-loyalty. However, only a positive relationship between perceived usefulness and e-loyalty was confirmed (**hypothesis H5**) in the proposed comprehensive model of online shopping behavior. A positive relationship between perceived ease of use and e-loyalty was not demonstrated (**hypothesis H6**). Also, a positive relationship between online shopping attitude and e-loyalty was not shown (**hypothesis H7**).

A positive relationship between perceived ease of use, perceived usefulness and attitude was demonstrated in Davis’s TAM model [18,25]. Surprisingly, this relationship between the mentioned factors in the comprehensive model of online shopping behavior was not proven (**hypothesis H8**), i.e. the relationship between the ease of use factor and perceived usefulness in the context of online shopping behavior was not proven, and also the positive relationship between perceived ease of use and attitude to online shopping was not proven (**hypothesis H9**). Furthermore, a positive relationship between perceived usefulness and intention to shop online was not demonstrated (**hypothesis H10**). A positive relationship was only shown between perceived usefulness and attitude towards online shopping (**hypothesis H11**), i.e. if online shoppers perceive online shopping as useful, then the likelihood of a positive attitude towards online shopping increases. The conflicting results of this research with the results of the TAM model [18,25] can be explained by its limits and shortcomings. In general, TAM focuses on the individual computer user, with the concept of "perceived usefulness", expanded to include other factors explaining how the user "perceives" "usefulness", thus ignoring the social processes of IS development and implementation. The perceived usefulness and ease of use framework overlooks other issues such as cost and structural requirements that drive users to adopt technology [114]. Legris et al. [115] claim that TAM and TAM2 together account for only 40% of technology system utilization.

The subjective norm factor was identified by the TRA model [26], or also by the C-TPB-TAM [20]. These models demonstrated a direct positive relationship between subjective norm and purchase intention. However, this positive relationship was not demonstrated in a comprehensive model of online shopping behavior (**hypothesis H13**). The subjective norm is determined by the perceived social pressure from others to behave in a certain way and their motivation to comply with those people’s opinions [82]. This perceived social pressure was not demonstrated for online shopping intention.

Perceived behavioral control was processed, for example, in the C-TAM-TPB model [20,29]. Noar and Zimmerman [86] demonstrated that perceived behavioral control not only influences actual behavior directly, but also influences it indirectly through behavioral intention. According to Taylor and Todd [29], behavioral intention is expected to fully mediate the relationship between perceived behavioral control and behavior. However, in a comprehensive model of online shopping behavior, a positive relationship between perceived behavioral control and intention to shop online was not demonstrated (**hypothesis H14**). Perceived behavioral control differs from other aspects and providing a person having different perceptions of behavioral control depending on the situation [116]. These different attitudes over perceived behavioral control were also found among online shoppers within a comprehensive model of online shopping behavior.

The relationship between attitude toward usability and behavioral intention was first demonstrated in the TRA model [85]. This relationship has been confirmed many times by other researches that dealt with behavioral models, especially in the field of online shopping behavior [9,46,53,78–80] This positive relationship between attitude and intention to online shopping was also clearly demonstrated in a comprehensive model of online shopping behavior (**hypothesis H15**).

The group of psychological factors includes the factors: congruence, perceived values, impulse buying behavior, shopping experience and shopping and browsing experience, as in the extension of Chisnall’s model [17]. In the context of online shopping, it can be expected that the congruence between the buyer, the technology (i.e. the Internet) and a particular product or service will be decisive in determining whether online shopping will be the preferred purchase channel for consumers [17]. However, the relationship between congruence and intention to shop online was not demonstrated in the comprehensive model of online shopping behavior, unlike Chisnall’s extended model [17]. The same conclusions were reached between shopping experience and intention to shop online. Venkatesh et al. [17] demonstrated a positive relationship in their model, but the proposed comprehensive model of online shopping behavior refuted this relationship. On the contrary, the comprehensive model of online shopping behavior confirmed a positive relationship between the perceived value factor, impulsive buying behavior, shopping experience and shopping and browsing enjoyment, and intention to online shopping. These results were also confirmed by the extended Chisnall model ([17]. Therefore, the **hypothesis H16** that psychological factors positively influence the intention to shop online cannot be unequivocally confirmed or refuted. Statistically, this hypothesis cannot be confirmed.

The research, like the extended Chisnall model [17], focused on time availability and time management. Research has examined the relationship between cultural factors and psychological factors. Venkatesh et al. [17] clearly confirmed the relationship between cultural and psychological factors, but the proposed comprehensive model of online shopping behavior did not confirm this relationship. The research evaluated that the relationship between cultural and psychological factors in the process of online shopping behavior cannot be clearly confirmed (**hypothesis H17**).

Furthermore, Venkatesh et al. [17] demonstrated a positive relationship between economic factors and psychological factors. However, the proposed comprehensive model of online shopping behavior did not confirm this relationship. It can therefore be evaluated that the psychological factors of the process of online shopping behavior are not influenced by economic factors. The group of demographic factors was also inspired by Chisnall’s extended model [17] taking into account mainly the age, gender, disposable income and education of Internet users. However, the influence of demographic factors on psychological factors in a comprehensive model of online shopping behavior has not been clearly demonstrated (**hypothesis H18**).

Previous studies have confirmed that intention to shop online plays an important role in predicting behavior [17,65,117], and this direct relationship has been demonstrated in many behavioral models [17,18,26]. Online shopping behavior also provides a comprehensive understanding of how antecedent variables influence outcomes, and we also examine support for the proposed model with behavior as the dependent variable. A strong positive relationship between intention and online shopping behavior was also confirmed in the comprehensive model of online shopping behavior (**hypothesis H19**).

## Conclusion

The research clearly showed that the key factors for customers when shopping online are Security and risk elimination (SE), together with the Online distribution and logistics (OD) and Online payments (OP) and E-shop certification (EC). Other important factors that influence the behavior of customers when purchasing online are: Webdesign and web orientation (WW), i.e. web design and orientation on the website, Online communication (OC), Online visualization and product description (OV), Customer service (CS). Research has confirmed that a wide range of offered goods is important for customers when shopping online.

On the contrary, the research shows that online shopping is not a dominant issue for customers (in general, shopping in brick-and-mortar stores still dominates). Multi-channel sales is also not a decisive factor for customers, i.e. it is not necessary for them to try the goods physically, even if they prefer this option when shopping online. Another surprising finding is the fact that lower price is not decisive for customers when shopping online, although it is still an important factor in online shopping behavior. Impulsive online shopping was identified by customers as the least important factor, which implies that online purchases tend to be more planned and thought out.

Based on previous extensive studies that have focused on the importance of various factors of online shopping, this paper aims to provide an integrative, comprehensive and nomological view of the issue of online shopping. To fulfill the main objective of the paper, a combination of basic and empirical research methods is used to develop and verify a comprehensive model of online shopping behavior. Such a model can help integrate the current diverse range of factors that have previously been studied piecemeal. Validation of this model in the context of real business practice with the results of consumer behavior makes it possible to gain comprehensive insight into the relative importance and relationships of these individual factors to online shopping behavior. Such a model can be used for further research and business practice. Online retailers can use the findings to better assess how the primary drivers of online shopping behavior affect their own business outcomes.

This paper presents a comprehensive, empirical investigation of the determinants of online shopping behavior among consumers in an online environment. The theoretical framework and empirical research for creating and validating a comprehensive model of online shopping behavior take an integrative, holistic approach to understanding the phenomenon of online shopping. The originality of the paper is based on the proposing the unique behavioral model that includes interrelationships between identified factors of online shopping behavior, thereby providing a comprehensive understanding of the investigated phenomenon. With the growing importance of online shopping, the proposed comprehensive model represents an integration of current knowledge and a basis for further research in the researched area. The validated model provides a comprehensive explanation of the current phenomenon of online shopping that integrates and extends previous studies identifying behavioral models of online shopping behavior. Further research will focus on identifying differences in online shoppers by age group and gender in the proposed comprehensive model of online shopping behavior. Furthermore, the research will focus on other target groups of respondents who come from another European region (from Visegrad countries), which will enable a relevant comparison and enable the possible validity of the findings of this research.

## Managerial implications and limits

The benefits of this study can also be seen as managerial implications. Online sellers can use the findings of this work to better assess the way in which the primary factors of online shopping behavior affect their own business results and can use them in determining their own e-commerce strategy. Validation of this model in the context of real business practice with the results of consumer behavior allows to gain comprehensive insight into the relative importance and relationships of these individual factors to online shopping behavior. The following conclusions therefore follow for online traders and marketers: The online customer must feel safe when shopping online, the trader must be trustworthy (ideally certified by APEK, etc. with good references about the products and services offered and the e-shop itself), the trader should place should offer a wide range of products and services in one place, it should also offer him a wide range of payment and transport options on the Internet and the possibility of multi-channel sales, furthermore, website visualization and user-friendly orientation on the website should be a priority for the merchant, together with clear information and visualization of the offered products and services and should not forget about customer care in the form of added value to the customer and offer him the possibility of communication and consultation online at a time that is convenient for the customer. All these factors positively influence the attitudes and intentions of customers towards online shopping and at the same time create a prerequisite for repeated online purchases. The combination of these factors, which the customer considers important, leads to his loyalty. A high percentage of loyal customers then creates a prerequisite for a stable position of an online merchant on B2C e-commerce markets and a long-term strengthening of its competitiveness.

The limits of empirical research can be summarized as follows. A certain limitation is that the subjects of the research were respondents from only one country of the European Union (Czech Republic). Despite the detailed and original methodology of primary research data collection, there are other methods that were able to ensure data collection using a different methodology (e.g. CAWI method). The other limitation could be based on the selected technique of data collection that could mispresent the results. The other limitation could be also based the period of gathering data, i.e. the period of increasing inflation, the perceived uncertainty resulting from the war conflict in Ukraine and thus the affected attitudes of European respondents.

Only one mathematical-statistical method PLS-SEM was applied to evaluate the formulated hypotheses. The possibility of verifying the achieved causal relationships between the defined factors, e.g. by implementing LRM (linear regression modeling) due to the scope was not used. Due to the robustness of the comprehensive model of online shopping behavior (see S1 Fig 2 in S1 File), it would be necessary to re-verify them on another sample set of respondents.

Respondents’ attitudes towards their online shopping behavior are largely limited by the customer’s current socio-economic situation. The subjectivity of attitudes is considerably sensitive in the context of increasing food, energy and inflation prices, which can change the customer’s view of selected attitudes to claims of online shopping behavior.

## Supporting information

S1 File (DOCX)

S1 Fig Model shaping the determinants of shopping behavior (Source: Own processing). (TIF)

S2 Fig Proposal of comprehensive model of online shopping behavior (Source: Own processing). (TIF)

S3 Fig Scree plot of final model (Source: Own processing). (TIF)

S4 Fig Evaluation of a comprehensive model of online shopping behavior (Source: Own processing). (TIF)
