# Supplementary material for: Modelling of online shopping behavior in the Czech online environment
Source: PLoS One. 2025 Jan 15;20(1):e0308725. doi: 10.1371/journal.pone.0308725 (PMC11734907; doi:10.1371/journal.pone.0308725)
Supplement: S1 File — (DOCX) [file pone.0308725.s001.docx]

**Supporting information 1: Theoretical background**

**S1 Table 1. Classification of models and theories used for online shopping behavior**

| **Theory and model classification** | | **Examples of theories and models** |
| --- | --- | --- |
| **Methods of development** | Extraction | TRA, MPCU, IDT, MM, SCT |
|  | Adoption | TPB, RRA, DTPB, TAM, TAM2, TAM3, C-TPB-TAM, UTAUT, UTAUT2 |
| **Scientific field** | Social psychology | TRA, TPB, DTPB, MM, RRA |
|  | Social sciences | IDT SCT |
|  | IT sciences | TAM, TAM2, C-TAM-TPB, MPCU |
|  | Combination of social psychology and IT sciences | UTAUT, UTAUT2, extended Chisnall model |

Source: own according to [24]

**S1 Table 2. Identification of determinants of online shopping behavior**

| **Determinant of online shopping behavior** | **Characterization of determinant of online shopping behavior** | **Sources** |
| --- | --- | --- |
| **E-security** | | |
| **Security and risk elimination for online sellers and buyers** | Compliance with e-commerce legislation, compliance with business terms and GDPR, use of modern technologies securing the e-shop, data protection and personal data certificates to increase the credibility and protection of the e-shop; Security of payment systems | Gefen et al., 2003  Roca et al., 2009  Perea y Monsuwé et al. (2004)  Mandal a Sur, 2017  Hasslinger et al., 2007  Kim a Park, 2013  Ijaz a Rhee, 2018  Svatošová, 2021  [9,10,32,43,45,66,108,109] |
| **Online payments** | The range of payment options on a specific e-shop and their number (their number and variability, i.e. bank transfer, in person, cash on delivery, payment gateway, payment aggregators, payment card, electronic wallets, installment purchase, m-payment), variability and offering more payment options on the Internet | APEK, 2022  Chen at al., 2019  Bucko et al., 2018  Mandal a Sur, 2017  Ijaz a Rhee, 2018  Svatošová, 2021  [5,9,10,49,82,108] |
| **Online distribution and logistics** | The speed of delivery of goods (time frame within 24 hours to 7 days) and the range of delivery options and their number (in person, by transport company, by post, pick-up points, so-called pick-up points, parcel shops, personal collection) – variability and offer more transport options | Chaffey et al., 2006  Mandal a Sur, 2017  APEK, 2022  Bucko et al., 2018  Chen at al., 2019  Svatošová, 2021  [5,49,82,108,110] |
| **E-trust** | | |
| **E-shop certification** | Whether or not the online business is certified (APEK certification or Verified by customers! from Heuréka.cz, or another form of certification); this factor is specific to the Czech online environment, for the online customer, store certification is not a condition, but it increases the overall credibility of the online seller | APEK, 2022  ČSÚ, 2022  Pilík, 2012  Pilík, 2015  Svatošová, 2021  [1,5,10,111,112] |
| **Product references** | Positive or negative customer references on social networks, or other discussion portals, or the absence of references about a selected sample of products offered online (or the main products of e-shops), or even offline; a factor that increases or decreases the overall credibility of an online seller | Bucko et al., 2018  Zhang et al., 2018,  Rosario et al., 2016  Venkatesh et al., 2022  Hasslinger et al., 2007  Svatošová, 2021  [10,17,45,49,81,113] |
| **E-shop references** | Positive or negative customer references on social networks, or other discussion portals, or the absence of references about the e-shop; a factor that increases or decreases the overall credibility of an online seller | Bucko et al, 2018  Zhang et al., 2018,  Rosario et al., 2016  Venkatesh et al., 2022  Hasslinger et al., 2007  Svatošová, 2021  [10,17,45,49,81,113] |
| **Trust in e-shop** | Provision of full contact and identification data in the e-shop, compliance with legal conditions associated with e-commerce (protection of personal data, compliance with the legal deadline for returning goods without giving reasons within 14 days, provision of business conditions, complaints procedure, negative or no customer reviews), delivery of goods in the required time and quality, added value to the online purchase, the customer evaluates the online seller as a serious e-shop that complies with all the conditions agreed between the seller and the buyer | Gefen et al., 2003  Ijaz a Rhee, 2018  Hsu et al., 2014,  Mccole et al., 2010,  Chen et al., 2019,  Al-Debei et al., 2015  Roca et al., 2009  Perea y Monsuwé et al. (2004)  Mandal a Sur, 2017  Hasslinger et al., 2007  Svatošová, 2021  [9,10,32,35,43,45,66,70,108,114] |
| **E-satisfaction** | | |
| **Websites and webdesign** | Compliance with the level of web design, the so-called F-display, which increases easy orientation for users in the e-shop and the probability of purchase, graphic design of e-shops, animation of the e-shop, good search structure - according to parameters, filters, etc., responsive design for all used devices (smartphones, tablets, laptops), clarity of information and user environment of the website; quality web design leads to higher satisfaction (e-satisfaction) | Bucko et al., 2018  Venkatesh et al., 2022  Chen at al., 2019  Mandal a Sur, 2017  Hasslinger et al., 2007  Svatošová, 2021  [10,17,45,49,82,108] |
| **Online communication** | Time zone defined by the online seller for communication with the customer (up to 7 days a week even after normal working hours), communication channels with the customer (social networks, online chat communication on the e-shop, Chatbots, telephone line, e-mail communication), satisfaction (e-satisfaction) of customers with the level of communication with the online seller | Kumar, 2018  Zhang et al., 2017,  Rosario et al., 2016 Venkatesh et al., 2022  Chen at al., 2019  Mandal a Sur, 2017  Svatošová, 2021  [10,17,81,82,108,113,115] |
| **Online visualization and product description** | Well-described technical information about the product, high-quality 2D and 3D images about the product, informational and instructional video about products, customer references about products directly on the e-shop website; this factor increases the probability of purchase, a part of e-shop promotion | Hsu et al, 2014  Venkatesh et al., 2022  Chen at al., 2008  Mandal a Sur, 2017  Svatošová, 2021  [10,17,70,82,108] |
| **Customer service** | Premium services for online shopping and their number (free shipping, extended product warranty, extended legal period for returning goods without giving reasons, i.e. more than 14 days, installation and assembly, return of goods to the floor); a factor that increases the likelihood of purchase and increases competitive advantage in the online environment | Bucko et al., 2018  Kumar, 2018  Chaffey et al., 2006  Mandal a Sur, 2017  Chen at al., 20019  Svatošová, 2021  [10,49,82,108,110,115] |
| **Multichannel sales** | The online seller sells only online in the e-shop, or has also set up one or more brick-and-mortar stores, where the customer has the opportunity to view and try the goods before online and offline purchase; a factor that is currently one of the deciding factors for online shopping | Bucko et al., 2018  Wareham et al., 2005,  Venkatesh et al., 2022  Chen at al., 2019  APEK, 2022  Svatošová, 2021  [5,10,17,49,82,116,117] |
| **General e-factors** | | |
| **Lower prices** | Lower price of products offered online compared to offline competition; Lower price of products offered online compared to online competition; Lowest price of products offered online in the entire online and offline market; the customer may prefer an online purchase to an offline purchase if the goods are cheaper in the online environment; can choose the cheapest comparable product through price comparators | Bucko et al., 2018  Pilík, 2012; Pilík, 2015  Pilík et al., 2017  Mandal a Sur, 2017  Hasslinger et al., 2007  Akbar et al., 2015  Chen at al., 2019  Chaffey et al., 2006  Svatošová, 2021  [10,45,49,51,52,82,108,110,111] |
| **Unlimited time and convenience of purchase** | The possibility of purchasing anytime and anywhere, 24 hours a day, 7 days a week, with unlimited purchases, other services are offered – advisory service, communication with the online seller, additional information about the product or services | Ijaz a Rhee, 2018  Al-Debei et al., 2015  Pilík, 2015  Pilík et al., 2017  APEK, 2022, Svatošová, 2021  Mandal a Sur, 2017  Hasslinger et al., 2007  Chen at al., 2019  Chaffey et al., 2006  [9,10,35,45,51,82,108,110,112] |
| **Wider assortment offer** | The breadth of the specific range of goods offered (enough brands, variants of the offered product, the possibility of choosing the offered goods, additional assortment, substitute products offered within one e-shop) | APEK, 2022  Akbar and James, 2014  Mandal a Sur, 2017  Chen at al., 2008  Svatošová, 2019  [5,10,52,82,108] |

Source: own processing

**S1 Table 3. Models/theories used for designing the comprehensive model of online shopping behavior**

| Model/Theories | Strengths | Weaknesses | Sources |
| --- | --- | --- | --- |
| TAM | It is a powerful model for technological applications. It replaced behavioral attitude in the TRA model with two measures of technology adoption, namely: perceived usefulness and perceived ease of use. It is less general than TRA and TPB. It provides feedback on two factors: usefulness and ease of use. | As an extension, TAM does not specify how expectations influence behavior. It also cannot predict user behavior within a culture; it thus ignores the social processes of IS development and implementation. | Davis, 1985  Davis, 1989  Taylor a Todd, 1995b Taylor a Todd, 1995c Venkatesh, 2000 Venkatesh a Davis, 2000  Momani et al., 2017  [20,24,25,25,28,29,38] |
| C-TAB-TPB | It combines the TPB model from the field of social psychology with the TAM from the field of information technology to better utilize the TPB in technology adoption. | TAM constructs do not fully reflect the specific influences of the context of use factor that can alter user acceptance. The behavioral planning factor is not taken into account. It still does not pay attention to the fear or threat regarding the use. | Ajzen a Fishbein, 1980  Davis, 1989  Taylor a Todd, 1995b  Momani et al., 2017  [18,19,24,26] |
| Extended Chisnall model | A comprehensive model combining the approaches of behavioral and informational models, takes into account external factors of online/offline shopping behavior, which may affect behavioral performance. | The model based on Chisnall's original model, which has its limits and only expands them with the constructs of online shopping behavior, it does not take into account the constructs verified in other behavioral and informational models in the context of online shopping behavior. | Svatošová, 2023; Venkatesh et al., 2022  [12,17] |

Source: own processing

**
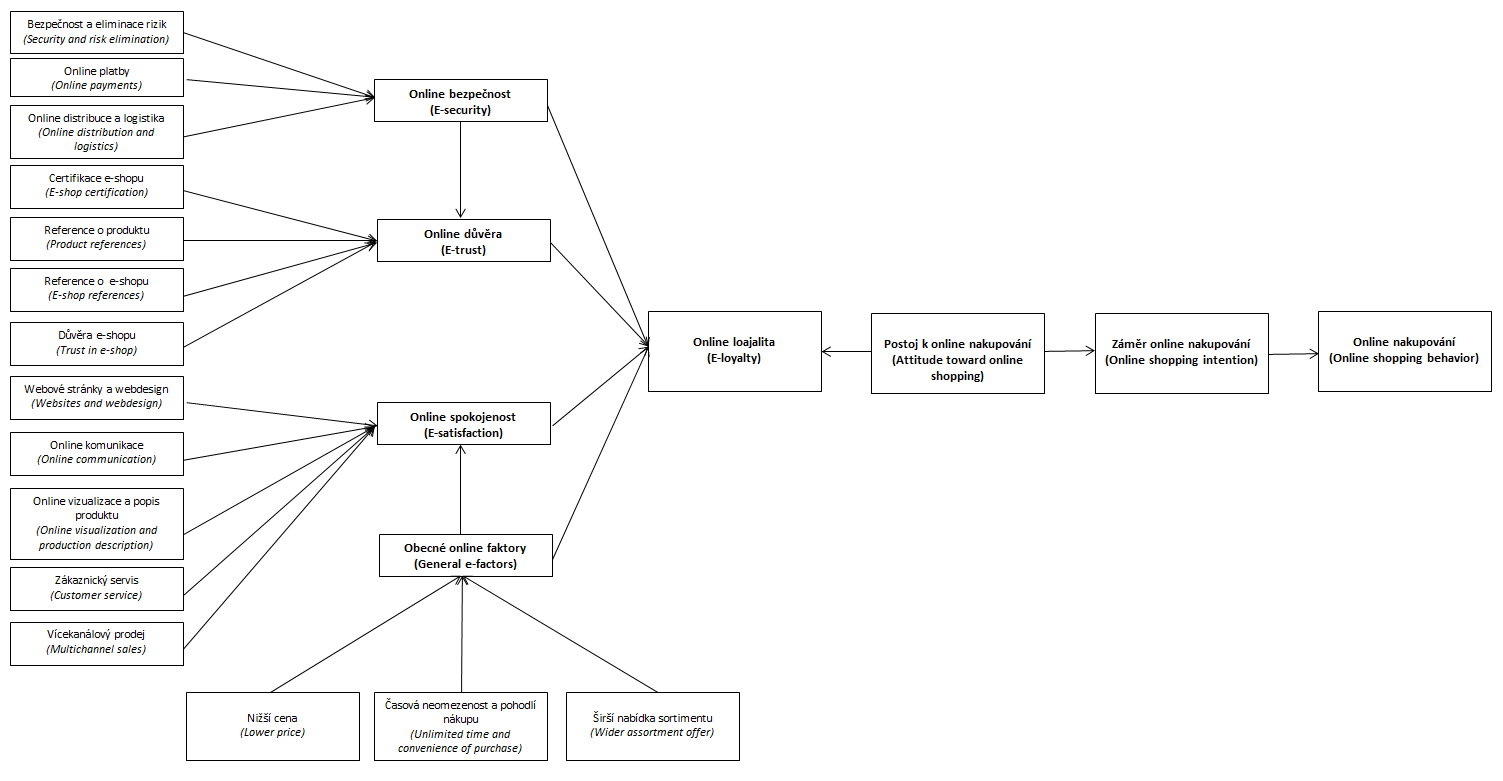
**

**S1 Fig 1. Model shaping the determinants of shopping behavior** (Source: own processing)


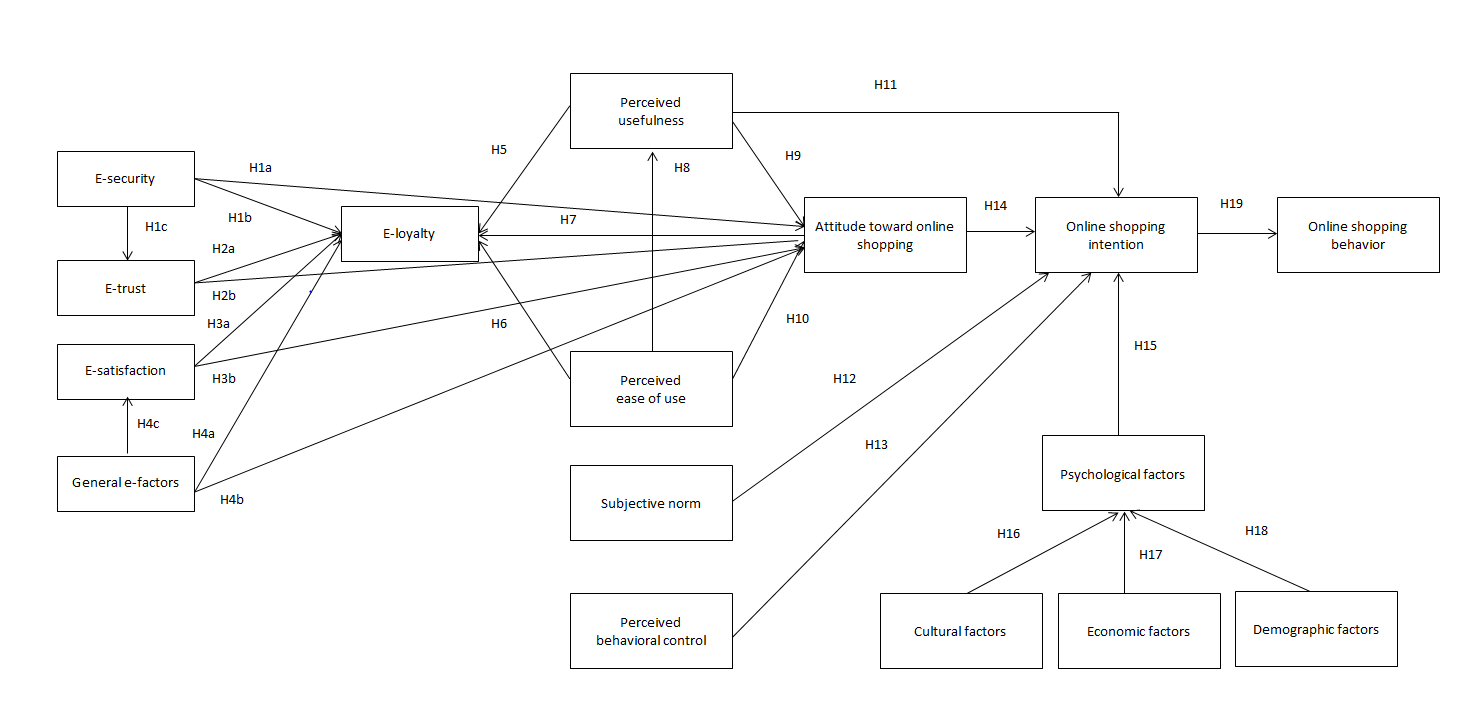


**S1 Fig 2. Proposal of comprehensive model of online shopping behavior** (Source: own processing)

# Supporting information 2: Variables definition and structure of respondents

S2 Table 4. Minimum, ideal and actual required number of respondents in each age category

| **Age group** | **Number of online shoppers according to CSO** | **The minimum required number of respondents in a questionnaire survey** | **The ideal required number of respondents in a questionnaire survey** | **The real number of respondents in a questionnaire survey** |
| --- | --- | --- | --- | --- |
| X: Generation Alpha (less than 15 years old) | **x** | **x** | x | x |
| A: The online generation (15 to 24 years old) | **11.11 %** | **42** | 85 | 264 |
| B: Millennials (25-39 years old) | **22.36 %** | **86** | 173 | 246 |
| C: Husák's children (40 to 54 years old) | **27.53 %** | **106** | 213 | 229 |
| D: Baby Boomers (ages 55-76) | **31.15 %** | **120** | 239 | 124 |
| E: War generation (over 77) | **7.85 %** | **31** | 60 | 63 |
| **In summary** | **100.00 %** | **385** | 770 | 926 |

Source: own processing

**Variables definition**

**Attitude toward online shopping (AT**): AT1: Online shopping suits me. AT2: I like to buy what I need in e-shops. AT3: I rate online shopping positively. **Online shopping intention (OSI)**: OSI1: I prefer to shop online rather than in physical stores. OSI2: I plan to shop more online in the future. OSI3: I will probably recommend online retailers where I have shopped in the past to my friends. OSI4: I plan to shop online in the next six months. **Online shopping behavior (OSB)**: OSB1: I regularly shop online. OSB2: I shop online a lot. OSB3: Overall, I have made many online purchases in the past. OSB4: On average, how often have you shopped online in the last six months? 0-1 times (1), 2-4 times (2); 5-7 times (3); 8-10 times (4); more than 11 times (5). OSB5: How many products do you usually buy per online purchase? 1 – 2 products (1), 3 – 4 products (2), 5 – 7 products (3), 8 – 10 products (4); more than 11 products. **Perceived usefulness (PU)**: PU1: I find online shopping useful when buying what I need. PU2: Online shopping improves my online shopping skills. PU3: Online shopping is comfortable, especially for easy search and purchase of products. PU4: Online shopping useful especially for easy search and purchase of products. **Perceived ease of use (PEU)**: PEU1: Most e-shops are user-friendly for online shopping. PEU2: Learning to shop online is easy. PEU3: I can easily find the product/service I'm looking for in most e-shops. PEU4: Online shopping makes it easier to compare products/services from different sellers. **Subjective norm (SN)**: SN1: People who are important to me support me in online shopping. SN2: People who influence me support me in online shopping. SN3: It is important to me what my surroundings (family, relatives, friends) think when I shop online. **Perceived behavioral control (PBC)**: PBC1: I have control over online shopping. PBC2: I always buy online only the products I intended. PBC3: I prefer online shopping because I can better control what I buy. **E-loyalty (L)**: L1: I regularly return to e-shops where I shopped online. L2: I usually buy products online in my favorite e-shop, even if it is more expensive than in other e-shops. L3: The main reason why I shop online is to buy products from my favorite brand that is online. **E-security: Security and risk elimination (SRE)**: SRE1: I feel safe when buying from e-shops that protect my privacy. SRE2: I trust a secure online retailer to protect my private information. SRE3: I am satisfied with buying what I want from secure online sellers. SRE4: I will only purchase a product online if I am convinced that the purchase I made fully meets my expectations. **Online payments(OP)**: OP1: I prefer shopping online because online sellers can offer me a payment method that suits me. OP2: I prefer shopping in an e-shop that offers a payment method that suits me. OP3: I don't shop online if the e-shop doesn't offer the payment method I want. **Online distribution and logistics (OD)**: OD1: I prefer shopping online, because online sellers can offer me a form of delivery of goods that suits me. OD2: I prefer shopping in an e-shop that offers me a method of delivery that suits me. OD3: I don't shop online if the e-shop doesn't offer me the delivery method I want. **E-satisfaction: Websites and webdesign (WW)**: WW1: I shop online in an e-shop if I can navigate the web well. WW2: I shop online in the e-shop if I can find complete information about the product I want. WW3: I shop online in an e-shop if the website (e-shop) is well designed. WW4: When shopping online, the web design of the e-shop is important to me. WW5: Good orientation on the website (e-shop) is important to me when shopping online. **Online communication (O**C): OC1: When buying online, online communication with the online seller is important to me. OC2: I do not shop online if the e-shop does not offer any form of online communication with the online seller (customer line, chatbots, prompt e-mail communication). OC3: I prefer online shopping in an e-shop with which I can communicate online (customer line, chatbots, prompt e-mail communication). **Online visualization and product description (OV)**: OV1: I shop online mainly because online sellers provide me with a larger range of products/services. OV2: They shop online mainly because it allows me to purchase specific products/services; OV3: Online product visualization is important to me when shopping online. OV4: When shopping online, product description is important to me. OV5: The quality of most of the products I buy online meets my expectations. **Customer service (CS)**: CS1: Customer service is important to me when shopping online (for example, free shipping, extended product warranty, extended legal period for returning goods without giving reasons, i.e. more than 14 days, installation and assembly, return of goods within floor). CS2: I don't shop in an e-shop that doesn't offer any customer service (for example, successful delivery). CS2: I do not shop in an e-shop that does not offer any customer service (for example, free shipping, extended product warranty, extended legal period for returning goods without giving reasons, i.e. more than 14 days, installation and assembly, return of goods to the floor). CS3: I prefer online shopping in an e-shop that offers customer service (for example, free shipping, extended product warranty, extended legal period for returning goods without giving reasons, i.e. more than 14 days, installation and assembly, return on goods to the floor). **Multichannel sales (MC)**: MC1: I prefer shopping in an e-shop, where I can try the goods before buying online (for example, in a brick-and-mortar store). MC2: I don't shop online unless the e-shop offers the possibility to try the product in a brick-and-mortar store. MC3: I prefer choosing a product online and buying a product offline in a physical store. **General e-factors: Lower prices (LP)**: LP1: Online shopping saves me money. LP2: Lower prices are the main reason I shop online. LP3: I don't shop online if it's more expensive to buy a product online than in a physical store. **Unlimited time and convenience of purchase (UT)**: UT1: Online shopping allows me to shop faster and saves me time. UT2: Online shopping is more convenient for me than shopping in a physical store. UT3: I shop online because I can shop whenever I want. UT4: I shop online because it's the most convenient way to shop. **Wider assortment offer (WAO)**: WAO1: I prefer an e-shop where I can choose from a wider assortment offer. WAO2: When shopping online, a wider range of products is important to me. WAO3: I do not shop in an e-shop that does not have a sufficient range of products for me. **E-trust: Trust in e-shop (TE)**: TE1: I generally have confidence in online shopping. TE2: I feel safe buying from online websites that protect my privacy. TE3: I prefer online shopping for products/services from secure online sellers. TE4: I trust that my personal information will be properly protected when I shop online. **E-shop certification (EC)**: EC1: I prefer online shopping in an e-shop that is certified (APEK, Heureka). EC2: E-shop certification is an important factor for online shopping. EC3: I never shop in an e-shop that is not certified. **Product references (PR)**: PR1: Online product references are an important factor for me if I shop online. PR2: I prefer to buy a product online that has some references than none. PR3: I do not buy the intended (planned) product online if it has bad references. **E-shop references (ER)**: ER1: E-shop references are important to me when shopping online. ER2: I prefer to buy from an e-shop that has some references than none. ER3: I don't shop at the intended (planned) e-shop if it has bad references. **Psychological factors: Congruence (C)**: C1: I am more likely to buy online if I have a clear idea about the product/service. C2: I am less likely to buy products/services online from an e-shop that I have to interact with to make the purchase. C3: I am more likely to buy a product/service online if it is well described (including accompanying video, audio). **Value consciousness (VC)**: VC1: When I buy products online, I want to be sure that this purchase will be worth my while. VC2: I generally buy products online at lower prices, but they still have to meet certain quality requirements before I buy them. VC3: I always check prices online to make sure I'm getting the best value for my money for the item I want. **Impulsive buyer behavior (IBB)**: IBB1: It often happens to me that when I buy goods online and then the goods are delivered to me, I was not sure afterwards why I bought them. IBB2: It often happens that I buy online, I don't care what product/service it is. IBB3: My cupboards are full of unused products I bought online. **Shopping experience (SE)**: SE1: I will not buy a product/service online in an e-shop that I do not know. SE2: I shop online mainly in e-shops where I have shopped in the past. SE3: As a rule, I visit a number of online stores before making the final online purchase of the desired product**. Shopping and browsing enjoyment (SBE**): SBE1: I shop online because I enjoy shopping online. SBE2: Online shopping is fun. SBE3: I shop online mainly to find the best product I want online. SBE4: I shop online mainly to buy goods at the best price. **Cultural factors: Time availability (TA)**: TA1: I am usually very busy with time. TA2: I don't have much free time. TA3: I find it difficult to find time for non-work related activities. **Time management (TM)**: TM1: I organize my time better than most people. TM2: I like to plan my activities according to time. TM3: Since I plan activities according to time, I can get more things done. Economic factors: **Objective consumption (OCO)**: OCO1: Before buying a product, it is important for me to know the guarantees of different brands. OCO2: Before buying a product, it is important for me to know the name of the company that manufactures the product. OCO3: Before buying a product, it is important for me to know if any brands are on sale. OCO4: Before purchasing a product, it is important to know the quality of the e-shop that offers the specific desired brand.

# Supporting information 3: Descriptive statistics of variables

**S3 Table 5. Descriptive characteristics of defined variables**

| **F** | **DF** | | | | **AT** | | | **OSI** | | | | **SBE** | | | | **TM** | | | **OSB** |
| --- | --- | --- | --- | --- | --- | --- | --- | --- | --- | --- | --- | --- | --- | --- | --- | --- | --- | --- | --- |
|  | **DF1** | **DF2** | **DF3** | **DF4** | **AT1** | **AT2** | **AT3** | **OSI1** | **OSI2** | **OSI3** | **OSI4** | **SBE1** | **SBE2** | **SBE3** | **SBE4** | **TM1** | **TM2** | **TM3** | **OSB1** |
| **M** | 2,434 | 1,538 | 2,894 | 2,801 | 3,737 | 3,708 | 3,919 | 2,779 | 3,702 | 3,681 | 4,134 | 3,029 | 2,308 | 4,257 | 4,353 | 3,631 | 4,030 | 4,104 | 3,518 |
| **SD** | 1,222 | 0,499 | 0,996 | 1,226 | 1,306 | 1,176 | 1,296 | 0,939 | 1,176 | 0,947 | 1,247 | 0,946 | 0,462 | 0,916 | 0,937 | 0,776 | 1,067 | 1,096 | 0,882 |
| **SKE** | -0,753 | -1,981 | -0,967 | -0,883 | -0,571 | -0,300 | -0,503 | 0,038 | -0,446 | 1,456 | 0,254 | -0,917 | -1,307 | 0,642 | 0,883 | 0,652 | -0,296 | -0,276 | 1,098 |
| **KUR** | 0,465 | -0,152 | -0,412 | 0,197 | -0,837 | -0,838 | -0,919 | -0,002 | -0,840 | -1,411 | -1,286 | 0,425 | 0,834 | -1,206 | -1,389 | -1,628 | -0,975 | -1,049 | -0,985 |
| **F** | **PU** | | | | **PEU** | | | | **SN** | | | **PBC** | | | **OCO** | | | | **OSB** |
|  | **PU1** | **PU2** | **PU3** | **PU4** | **PEU1** | **PEU2** | **PEU3** | **PEU4** | **SN1** | **SN2** | **SN3** | **PBC1** | **PBC2** | **PBC3** | **OCO1** | **OCO2** | **OCO3** | **OCO4** | **OSB2** |
| **M** | 3,956 | 3,079 | 3,728 | 3,005 | 2,755 | 2,626 | 3,180 | 3,577 | 3,863 | 3,778 | 2,473 | 4,285 | 4,174 | 2,952 | 4,144 | 2,502 | 2,614 | 4,307 | 3,262 |
| **SD** | 1,000 | 1,160 | 1,054 | 1,168 | 1,121 | 1,231 | 1,107 | 1,039 | 1,150 | 1,128 | 1,058 | 0,935 | 0,905 | 1,033 | 0,839 | 0,538 | 0,525 | 0,891 | 0,858 |
| **SKE** | 0,626 | -1,098 | -0,311 | -1,210 | -0,582 | -0,972 | -0,830 | 0,307 | 0,550 | 0,481 | 0,010 | 2,285 | 2,181 | -0,748 | 1,349 | -1,092 | -1,131 | 1,401 | 0,788 |
| **KUR** | -1,087 | 0,266 | -0,795 | 0,466 | 0,120 | 0,227 | -0,279 | -1,098 | -1,166 | -1,123 | 0,763 | -1,613 | -1,473 | 0,572 | -1,210 | 0,368 | -0,064 | -1,421 | -0,264 |
| **F** | **SE** | | | | **OP** | | | **OD** | | | **L** | | | **WW** | | | | | **OSB** |
|  | **SE1** | **SE2** | **SE3** | **SE4** | **OP1** | **OP2** | **OP3** | **OD1** | **OD2** | **OD3** | **L1** | **L2** | **L3** | **WW1** | **WW2** | **WW3** | **WW4** | **WW5** | **OSB3** |
| **M** | 4,238 | 4,156 | 4,090 | 4,081 | 4,262 | 4,138 | 3,963 | 4,449 | 4,261 | 3,997 | 3,997 | 3,634 | 2,676 | 4,150 | 4,091 | 3,217 | 3,254 | 4,070 | 3,177 |
| **SD** | 0,971 | 0,945 | 0,918 | 0,914 | 0,948 | 0,908 | 0,817 | 0,907 | 0,877 | 0,756 | 1,122 | 1,132 | 0,863 | 0,914 | 0,889 | 1,133 | 1,147 | 0,879 | 0,961 |
| **SKE** | 0,827 | 0,760 | 0,803 | 0,816 | 0,823 | 0,747 | 1,251 | 1,941 | 1,239 | 1,963 | 0,451 | -0,513 | -0,034 | 0,700 | 0,763 | -1,472 | -1,468 | 0,803 | 0,232 |
| **KUR** | -1,328 | -1,224 | -1,170 | -1,165 | -1,315 | -1,154 | -1,138 | -1,712 | -1,315 | -1,212 | -1,139 | -0,695 | 0,545 | -1,153 | -1,105 | 0,179 | 0,173 | -1,095 | -0,287 |
| **F** | **WAO** | | | **EC** | | | **PR** | | | **ER** | | | **TA** | | | **SE** | | | **OSB** |
|  | **WAO1** | **WAO2** | **WAO3** | **EC1** | **EC2** | **EC3** | **PR1** | **PR2** | **PR3** | **ER1** | **ER2** | **ER3** | **TA1** | **TA2** | **TA3** | **SE1** | **SE2** | **SE3** | **OSB4** |
| **M** | 4,069 | 3,311 | 3,119 | 4,334 | 4,040 | 3,437 | 3,901 | 3,319 | 3,543 | 4,245 | 3,441 | 3,961 | 2,032 | 2,532 | 2,126 | 2,944 | 3,785 | 3,812 | 2,591 |
| **SD** | 1,052 | 0,944 | 0,883 | 0,832 | 0,712 | 1,010 | 0,903 | 0,670 | 0,804 | 1,060 | 1,047 | 0,948 | 0,903 | 0,785 | 0,941 | 1,070 | 0,967 | 0,958 | 1,554 |
| **SKE** | 1,401 | -0,212 | 0,115 | 2,068 | 2,914 | -1,054 | -0,067 | -0,770 | -0,200 | 0,232 | -1,228 | 0,207 | -1,774 | -0,216 | -1,824 | -0,848 | 0,659 | 0,895 | -1,278 |
| **KUR** | -1,389 | -0,455 | -0,092 | -1,503 | -1,352 | 0,190 | -0,747 | -0,474 | -1,292 | -1,265 | -0,128 | -0,992 | -0,064 | -1,248 | -0,254 | 0,245 | -0,992 | -1,087 | 0,545 |

| **F** | **C** | | | **VC** | | | **IBB** | | | **OV** | | | | | **UT** | | | | **OSB** |
| --- | --- | --- | --- | --- | --- | --- | --- | --- | --- | --- | --- | --- | --- | --- | --- | --- | --- | --- | --- |
|  | **C1** | **C2** | **C3** | **VC1** | **VC2** | **VC3** | **IBB1** | **IBB2** | **IBB3** | **OV1** | **OV2** | **OV3** | **OV4** | **OV5** | **UT1** | **UT2** | **UT3** | **UT4** | **OSB5** |
| **M** | 3,382 | 3,102 | 4,056 | 3,021 | 2,819 | 3,530 | 1,849 | 1,793 | 1,590 | 4,286 | 4,551 | 4,252 | 3,961 | 4,302 | 3,719 | 3,635 | 3,576 | 3,165 | 2,519 |
| **SD** | 0,630 | 0,501 | 0,730 | 0,950 | 0,542 | 0,601 | 0,929 | 0,892 | 0,819 | 0,857 | 0,882 | 0,847 | 0,699 | 0,861 | 1,181 | 1,038 | 1,071 | 0,993 | 1,563 |
| **SKE** | -0,645 | 0,755 | 2,663 | -1,893 | -0,020 | -0,202 | -1,775 | -1,615 | -0,933 | 1,622 | 3,171 | 1,582 | 3,020 | 1,652 | -0,215 | 0,813 | 0,229 | 0,168 | -1,268 |
| **KUR** | -0,513 | 0,191 | -1,323 | -0,041 | -0,100 | -0,893 | 0,304 | 0,418 | 0,883 | -1,401 | -2,066 | -1,348 | -1,447 | -1,429 | -0,803 | -1,232 | -0,920 | -0,037 | 0,580 |
| **F** | **CS** | | | **MC** | | | **LP** | | | **OC** | | | **TE** | | | |  | | |
|  | **CS1** | **CS2** | **CS3** | **MC1** | **MC2** | **MC3** | **LP1** | **LP2** | **LP3** | **OC1** | **OC2** | **OC3** | **TE1** | **TE2** | **TE3** | **TE4** |  |  |  |
| **M** | 4,237 | 3,976 | 4,355 | 2,597 | 2,704 | 3,431 | 2,784 | 2,971 | 3,368 | 4,006 | 3,870 | 4,148 | 3,188 | 4,241 | 3,896 | 2,909 |  |  |  |
| **SD** | 0,906 | 0,785 | 0,932 | 0,774 | 0,884 | 1,075 | 0,869 | 0,994 | 0,990 | 1,011 | 0,958 | 1,091 | 0,810 | 0,839 | 0,638 | 0,876 |  |  |  |
| **SKE** | 0,789 | 1,382 | 1,067 | -0,283 | -0,751 | -0,090 | -0,539 | -1,020 | 0,297 | -0,356 | -0,217 | -0,390 | -0,880 | 1,511 | 4,128 | -0,970 |  |  |  |
| **KUR** | -1,218 | -1,089 | -1,435 | -0,271 | -0,129 | -0,948 | -0,330 | -0,451 | -1,304 | -0,843 | -0,797 | -1,012 | -0,025 | -1,303 | -1,789 | 0,438 |  |  |  |

*Source: own processing*

**S3 Table 6. Reliability and validity of selected factors in the questionnaire**

| **AT:** CA = 0.968 ; CR = 0.980; AVE = 0.941; KMO = 0.777 | | | | **PBC:** CA = 0.702 ; CR = 0.862; AVE = 0.647; KMO = 0.556 | | | | **OP:** CA = 0.926 ; CR = 0.954; AVE = 0.874; KMO = 0.742 | | | | **OC:** CA = 0.955 ; CR = 0.972; AVE = 0.922; KMO = 0.756 | | | |
| --- | --- | --- | --- | --- | --- | --- | --- | --- | --- | --- | --- | --- | --- | --- | --- |
|  |  |  |  |  |  |  |  |  |  |  |  |  |  |  |  |
| **I** | **CI-TC** | **FL** | **C** | **I** | **CI-TC** | **FL** | **C** | **I** | **CI-TC** | **FL** | **C** | **I** | **CI-TC** | **FL** | **C** |
| **AT1** | 0.921 | 0.956 | 0.930 | **PBC1** | 0,736 | 0,899 | 0,807 | **OP1** | 0,856 | 0,936 | 0,876 | **OC1** | 0,933 | 0,972 | 0,945 |
| **AT2** | 0.932 | 0.970 | 0.940 | **PBC2** | 0,767 | 0,929 | 0,862 | **OP2** | 0,894 | 0,954 | 0,911 | **OC2** | 0,915 | 0,964 | 0,929 |
| **AT3** | 0.946 | 0.976 | 0.953 | **PBC3** | 0,376 | 0,521 | 0,272 | **OP3** | 0,810 | 0,913 | 0,834 | **OC3** | 0,877 | 0,944 | 0,891 |
| **SN:** CA = 0.776 ; CR = 0.871; AVE = 0.701; KMO = 0.577 | | | | **L:** CA = 0.781 ; CR = 0.871; AVE = 0.696; KMO = 0.603 | | | | **OD:** CA = 0.947 ; CR = 0.968; AVE = 0.909; KMO = 0.759 | | | | **CS:** CA = 0.912 ; CR = 0.947; AVE = 0.855; KMO = 0.759 | | | |
|  |  |  |  |  |  |  |  |  |  |  |  |  |  |  |  |
| **I** | **CI-TC** | **FL** | **C** | **I** | **CI-TC** | **FL** | **C** | **I** | **CI-TC** | **FL** | **C** | **I** | **CI-TC** | **FL** | **C** |
| **SN1** | 0,736 | 0,923 | 0,852 | **L1** | 0,679 | 0,871 | 0,759 | **OD1** | 0,895 | 0,953 | 0,908 | **CS1** | 0,841 | 0,891 | 0,867 |
| **SN2** | 0,767 | 0,934 | 0,872 | **L2** | 0,767 | 0,916 | 0,838 | **OP2** | 0,922 | 0,966 | 0,933 | **CS2** | 0,830 | 0,940 | 0,856 |
| **SN3** | 0,376 | 0,713 | 0,375 | **L3** | 0,553 | 0,700 | 0,590 | **OP3** | 0,868 | 0,941 | 0,885 | **CS3** | 0,817 | 0,896 | 0,843 |
| **MC:** CA = 0.881 ; CR = 0.938; AVE = 0.835; KMO = 0.670 | | | | **WAO:** CA = 0.901 ; CR = 0.929; AVE = 0.814; KMO = 0.716 | | | | **PR:** CA = 0.777 ; CR = 0.871; AVE = 0.693; KMO = 0.633 | | | | **C:** CA = 0.810 ; CR = 0.894; AVE = 0.739; KMO = 0.595 | | | |
|  |  |  |  |  |  |  |  |  |  |  |  |  |  |  |  |
| **I** | **CI-TC** | **FL** | **C** | **I** | **CI-TC** | **FL** | **C** | **I** | **CI-TC** | **FL** | **C** | **I** | **CI-TC** | **FL** | **C** |
| **MC1** | 0,867 | 0,891 | 0,916 | **WAO1** | 0,665 | 0,833 | 0,694 | **PR1** | 0,733 | 0,900 | 0,810 | **C1** | 0,818 | 0,936 | 0,877 |
| **MC2** | 0,839 | 0,940 | 0,904 | **WAO2** | 0,828 | 0,937 | 0,878 | **PR2** | 0,528 | 0,766 | 0,587 | **C2** | 0,602 | 0,824 | 0,679 |
| **MC3** | 0,661 | 0,896 | 0,684 | **WAO3** | 0,821 | 0,932 | 0,869 | **PR3** | 0,613 | 0,826 | 0,681 | **C3** | 0,616 | 0,813 | 0,662 |
| **LP:** CA = 0.956 ; CR = 0.940; AVE = 0.840; KMO = 0.766 | | | | **EC:** CA = 0.848 ; CR = 0.921; AVE = 0.797; KMO = 0.551 | | | | **ER:** CA = 0.875 ; CR = 0.928; AVE = 0.812; KMO = 0.523 | | | | **VC:** CA = 0.310 ; CR = 0.603; AVE = 0.593; KMO = 0.515 | | | |
|  |  |  |  |  |  |  |  |  |  |  |  |  |  |  |  |
| **I** | **CI-TC** | **FL** | **C** | **I** | **CI-TC** | **FL** | **C** | **I** | **CI-TC** | **FL** | **C** | **I** | **CI-TC** | **FL** | **C** |
| **LP1** | 0,858 | 0,942 | 0,887 | **EC1** | 0,682 | 0,847 | 0,770 | **ER1** | 0,718 | 0,888 | 0,810 | **VC1** | 0,203 | 0,425 | 0,180 |
| **LP2** | 0,841 | 0,937 | 0,879 | **EC2** | 0,904 | 0,627 | 0,936 | **ER2** | 0,620 | 0,814 | 0,587 | **VC2** | 0,296 | 0,877 | 0,769 |
| **LP3** | 0,726 | 0,868 | 0,754 | **EC3** | 0,635 | 0,887 | 0,684 | **ER3** | 0,975 | 0,992 | 0,681 | **VC3** | 0,141 | 0,910 | 0,828 |
| **IBB:** CA = 0.015 ; CR = 0.637; AVE = 0.495; KMO = 0.488 | | | | **SE:** CA = 0.830 ; CR = 0.907; AVE = 0.769; KMO = 0.544 | | | | **TA:** CA = 0.279 ; CR = 0.570; AVE = 0.379; KMO = 0.332 | | | | **TM:** CA = 0.966 ; CR = 0.984; AVE = 0.954; KMO = 0.755 | | | |
|  |  |  |  |  |  |  |  |  |  |  |  |  |  |  |  |
| **I** | **CI-TC** | **FL** | **C** | **I** | **CI-TC** | **FL** | **C** | **I** | **CI-TC** | **FL** | **C** | **I** | **CI-TC** | **FL** | **C** |
| **IBB1** | 0,542 | 0,923 | 0,851 | **SE1** | 0,460 | 0,675 | 0,455 | **TA1** | 0,117 | 0,934 | 0,953 | **TM1** | 0,916 | 0,962 | 0,925 |
| **IBB2** | 0,511 | 0,782 | 0,611 | **SE2** | 0,849 | 0,969 | 0,939 | **TA2** | 0,645 | 0,493 | 0,903 | **TM2** | 0,965 | 0,982 | 0,965 |
| **IBB3** | 0,384 | -0,670 | 0,449 | **SE3** | 0,809 | 0,955 | 0,913 | **TA3** | 0,123 | 0,146 | 0,886 | **TM3** | 0,969 | 0,985 | 0,970 |
| **OSI:** CA = 0.908 ; CR = 0.938; AVE = 0.793; KMO = 0.781 | | | | **PEU:** CA = 0.916 ; CR = 0.941; AVE = 0.801; KMO = 0.780 | | | | **UT:** CA = 0.878 ; CR = 0.970; AVE = 0.889; KMO = 0.692 | | | | **SBE:** CA = 0.678 ; CR = 0.560; AVE = 0.297; KMO = 0.476 | | | |
|  |  |  |  |  |  |  |  |  |  |  |  |  |  |  |  |
| **I** | **CI-TC** | **FL** | **C** | **I** | **CI-TC** | **FL** | **C** | **I** | **CI-TC** | **FL** | **C** | **I** | **CI-TC** | **FL** | **C** |
| **OSI1** | 0,595 | 0,741 | 0,549 | **PEU1** | 0,787 | 0,877 | 0,769 | **UT1** | 0,864 | 0,924 | 0,853 | **SBE1** | 0,408 | 0,515 | 0,632 |
| **OSI2** | 0,906 | 0,951 | 0,905 | **PEU2** | 0,854 | 0,921 | 0,848 | **UT2** | 0,932 | 0,963 | 0,928 | **SBE2** | 0,114 | 0,890 | 0,815 |
| **OSI3** | 0,888 | 0,942 | 0,887 | **PEU3** | 0,855 | 0,924 | 0,854 | **UT3** | 0,883 | 0,937 | 0,879 | **SBE3** | 0,724 | 0,156 | 0,899 |
| **OSI4** | 0,827 | 0,911 | 0,829 | **PEU4** | 0,748 | 0,856 | 0,733 | **UT4** | 0,908 | 0,947 | 0,897 | **SBE4** | 0,626 | 0,330 | 0,942 |
| **PU:** CA = 0.869 ; CR = 0.911; AVE = 0.720; KMO = 0.708 | | | | **SRE:** CA = 0.951 ; CR = 0.964; AVE = 0.872; KMO = 0.851 | | | | **TE:** CA = 0.767 ; CR = 0.864; AVE = 0.617; KMO = 0.537 | | | | **OCO:** CA = 0.168 ; CR = 0.186; AVE = 0.336; KMO = 0.282 | | | |
|  |  |  |  |  |  |  |  |  |  |  |  |  |  |  |  |
| **I** | **CI-TC** | **FL** | **C** | **I** | **CI-TC** | **FL** | **C** | **I** | **CI-TC** | **FL** | **C** | **I** | **CI-TC** | **FL** | **C** |
| **PU1** | 0,699 | 0,839 | 0,704 | **SRE1** | 0,883 | 0,935 | 0,874 | **TE1** | 0,691 | 0,847 | 0,718 | **OCO1** | 0,916 | -0,450 | 0,929 |
| **PU2** | 0,737 | 0,851 | 0,723 | **SRE2** | 0,890 | 0,939 | 0,882 | **TE2** | 0,360 | 0,627 | 0,393 | **OCO2** | 0,965 | 0,789 | 0,813 |
| **PU3** | 0,735 | 0,864 | 0,747 | **SRE3** | 0,889 | 0,939 | 0,882 | **TE3** | 0,786 | 0,887 | 0,787 | **OCO3** | 0,969 | 0,589 | 0,769 |
| **PU4** | 0,721 | 0,840 | 0,706 | **SRE4** | 0,860 | 0,921 | 0,848 | **TE4** | 0,522 | 0,756 | 0,571 | **OCO4** | 0,626 | -0,414 | 0,859 |
| **OSB:** CA = 0.929 ; CR = 0.961; AVE = 0.832; KMO = 0.793 | | | | **WW:** CA = 0.910 ; CR = 0.938; AVE = 0.748; KMO = 0.733 | | | | **OV:** CA = 0.947 ; CR = 0.961; AVE = 0.832; KMO = 0.880 | | | |  | | | |
|  |  |  |  |  |  |  |  |  |  |  |  |  |  |  |  |
| **I** | **CI-TC** | **FL** | **C** | **I** | **CI-TC** | **FL** | **C** | **I** | **CI-TC** | **FL** | **C** |  |  |  |  |
| **OSB1** | 0,727 | 0,841 | 0,707 | **WW1** | 0,811 | 0,907 | 0,822 | **OV1** | 0,830 | 0,891 | 0,794 |  |  |  |  |
| **OSB2** | 0,902 | 0,956 | 0,913 | **WW2** | 0,849 | 0,928 | 0,862 | **OV2** | 0,904 | 0,940 | 0,884 |  |  |  |  |
| **OSB3** | 0,881 | 0,937 | 0,879 | **WW3** | 0,774 | 0,830 | 0,689 | **OV3** | 0,835 | 0,896 | 0,803 |  |  |  |  |
| **OSB4** | 0,899 | 0,916 | 0,840 | **WW4** | 0,779 | 0,834 | 0,696 | **OV4** | 0,889 | 0,931 | 0,867 |  |  |  |  |
| **OSB5** | 0,886 | 0,908 | 0,824 | **WW5** | 0,707 | 0,820 | 0,673 | **OV5** | 0,843 | 0,901 | 0,812 |  |  |  |  |

Source: own processing

# Supporting information 4: KMO and Bartlett´s test of final model and Total variance explained (TVE) of final model

**S4 Table 7. Results of KMO and Bartlett's sphericity test**

| **Kaiser-Meyer-Olkin Measure of Sampling Adequacy (KMO)** | | 0,870 |
| --- | --- | --- |
| **Bartlett´s test of Sphericity** | **Approx. Chi-Square** | 81899,929 |
|  | **Df** | 4753 |
|  | **Sig. (p-value)** | 0,000^***^ |

Note: ^***^ α = 0.001. Source: own processing

**S4 Table 8. Explanation of the total variability of the defined factors in the comprehensive model of online shopping behavior**

| **Total Variance Explained** | | | | | | | | | |
| --- | --- | --- | --- | --- | --- | --- | --- | --- | --- |
| **F** | **Initial Eigenvalues** | | | **Extraction Sums of Squared Loadings** | | | **Rotation Sums of Squared Loadings** | | |
|  | **Total** | **% of Var.** | **Cum.**  **%** | **Total** | **% of**  **Var.** | **Cum.**  **%** | **Total** | **% of**  **Var.** | **Cum.**  **%** |
| **OSB** | 4,388 | 4,478 | 4,478 | 4,388 | 4,478 | 4,478 | 4,201 | 4,287 | 4,287 |
| **OV** | 4,353 | 4,442 | 8,919 | 4,353 | 4,442 | 8,919 | 4,186 | 4,271 | 8,558 |
| **UT** | 4,091 | 4,174 | 13,094 | 4,091 | 4,174 | 13,094 | 3,786 | 3,863 | 12,421 |
| **SRE** | 3,880 | 3,959 | 17,053 | 3,880 | 3,959 | 17,053 | 3,584 | 3,657 | 16,078 |
| **OSI** | 3,626 | 3,700 | 20,753 | 3,626 | 3,700 | 20,753 | 3,509 | 3,581 | 19,659 |
| **PEU** | 3,345 | 3,413 | 24,167 | 3,345 | 3,413 | 24,167 | 3,225 | 3,291 | 22,950 |
| **WW** | 3,232 | 3,298 | 27,465 | 3,232 | 3,298 | 27,465 | 3,197 | 3,262 | 26,212 |
| **TM** | 3,159 | 3,224 | 30,689 | 3,159 | 3,224 | 30,689 | 2,915 | 2,974 | 29,186 |
| **AT** | 3,102 | 3,166 | 33,854 | 3,102 | 3,166 | 33,854 | 2,876 | 2,935 | 32,121 |
| **OC** | 3,053 | 3,115 | 36,969 | 3,053 | 3,115 | 36,969 | 2,842 | 2,900 | 35,022 |
| **OD** | 2,956 | 3,016 | 39,985 | 2,956 | 3,016 | 39,985 | 2,810 | 2,867 | 37,889 |
| **PU** | 2,774 | 2,831 | 42,816 | 2,774 | 2,831 | 42,816 | 2,746 | 2,802 | 40,691 |
| **OP** | 2,680 | 2,735 | 45,551 | 2,680 | 2,735 | 45,551 | 2,646 | 2,700 | 43,391 |
| **ER** | 2,633 | 2,687 | 48,238 | 2,633 | 2,687 | 48,238 | 2,585 | 2,638 | 46,029 |
| **CS** | 2,590 | 2,643 | 50,881 | 2,590 | 2,643 | 50,881 | 2,538 | 2,589 | 48,618 |
| **MC** | 2,451 | 2,501 | 53,382 | 2,451 | 2,501 | 53,382 | 2,524 | 2,575 | 51,194 |
| **LP** | 2,435 | 2,484 | 55,866 | 2,435 | 2,484 | 55,866 | 2,467 | 2,517 | 53,711 |
| **EC** | 2,261 | 2,308 | 58,174 | 2,261 | 2,308 | 58,174 | 2,458 | 2,508 | 56,219 |
| **WAO** | 2,241 | 2,286 | 60,460 | 2,241 | 2,286 | 60,460 | 2,424 | 2,473 | 58,692 |
| **C** | 2,191 | 2,236 | 62,696 | 2,191 | 2,236 | 62,696 | 2,252 | 2,298 | 60,990 |
| **SE** | 2,093 | 2,136 | 64,832 | 2,093 | 2,136 | 64,832 | 2,247 | 2,293 | 63,282 |
| **TE** | 2,026 | 2,068 | 66,900 | 2,026 | 2,068 | 66,900 | 2,112 | 2,155 | 65,438 |
| **L** | 1,971 | 2,011 | 68,910 | 1,971 | 2,011 | 68,910 | 2,103 | 2,146 | 67,583 |
| **SBE** | 1,922 | 1,961 | 70,871 | 1,922 | 1,961 | 70,871 | 2,036 | 2,078 | 69,661 |
| **PR** | 1,823 | 1,860 | 72,731 | 1,823 | 1,860 | 72,731 | 2,015 | 2,057 | 71,718 |
| **TA** | 1,731 | 1,766 | 74,497 | 1,731 | 1,766 | 74,497 | 1,898 | 1,937 | 73,655 |
| **SN** | 1,683 | 1,717 | 76,214 | 1,683 | 1,717 | 76,214 | 1,893 | 1,931 | 75,586 |
| **OCO** | 1,625 | 1,658 | 77,872 | 1,625 | 1,658 | 77,872 | 1,797 | 1,833 | 77,420 |
| **PBC** | 1,560 | 1,592 | 79,465 | 1,560 | 1,592 | 79,465 | 1,708 | 1,743 | 79,162 |
| **IBB** | 1,552 | 1,584 | 81,049 | 1,552 | 1,584 | 81,049 | 1,685 | 1,719 | 80,881 |
| **VC** | 1,447 | 1,476 | 82,525 | 1,447 | 1,476 | 82,525 | 1,611 | 1,643 | 82,525 |

Note: % of Var. – Variance; Cum. % – Cumulative; F – Factor; Extraction Method – Principal Component analysis; Rotation: Varimax; Rotation converged in 14 iterations. Source: own processing


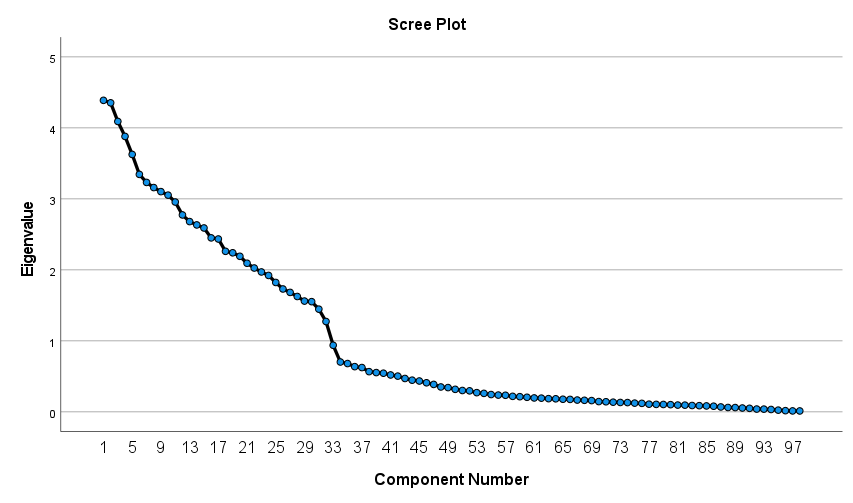


# S4 Figure 3. Scree plot of final model (Source: own processing)

# Supporting information 5: Regression estimation and verification of hypotheses of empirical research

**S5 Table 9. Estimation and verification of formulated hypotheses**

| **Sign**  **of H** | | **Relationships** | **Path Coef.**  **(unstand.)** | **SE** | **CR** | **Sig.**  **p-value** | **Evaluation** |
| --- | --- | --- | --- | --- | --- | --- | --- |
| H1a | H1a_1 | SRE >> L | 0.069 | 0.040 | 1.739 | 0.082 | Rejected |
|  | H1a_2 | OP >> L | 0.015 | 0.025 | 0.590 | 0.555 | Rejected |
|  | H1a_3 | OD >> L | 0.033 | 0.029 | 1.130 | 0.259 | Rejected |
| H1b | H1b_1 | SRE >> AT | 0.158 | 0.041 | 3.855 | 0.000^***^ | Supported |
|  | H1b_2 | OP >> AT | 0.179 | 0.044 | 4.069 | 0.000^***^ | Supported |
|  | H1b_3 | OD >> AT | 0.145 | 0.042 | 0.345 | 0.000^***^ | Supported |
| H1c | H1c_1 | SRE >> TE | 0.094 | 0.024 | 3.917 | 0.000^***^ | Supported |
|  | H1c_2 | OP >> TE | 0.099 | 0.027 | 3.667 | 0.000^***^ | Rejected |
|  | H1c_3 | OD >> TE | 0.009 | 0.019 | 0.474 | 0.632 | Rejected |
|  | H1c_4 | SRE >> EC | 0.003 | 0.016 | 0.195 | 0.845 | Rejected |
|  | H1c_5 | OP >> EC | 0.024 | 0.019 | 1.283 | 0.200 | Rejected |
|  | H1c_6 | OD >> EC | 0.073 | 0.032 | 2.301 | 0.021^*^ | Supported |
|  | H1c_7 | SRE >> RP | 0.006 | 0.015 | 0.391 | 0.696 | Rejected |
|  | H1c_8 | OP >> RP | 0.003 | 0.015 | 0.182 | 0.855 | Rejected |
|  | H1c_9 | OD >> RP | 0.036 | 0.036 | 1.025 | 0.305 | Rejected |
|  | H1c_10 | SRE >> ER | 0.021 | 0.027 | 0.781 | 0.435 | Rejected |
|  | H1c_11 | OP >> ER | 0.033 | 0.031 | 1.081 | 0.280 | Rejected |
|  | H1c_12 | OD >> ER | 0.053 | 0.037 | 1.433 | 0.152 | Rejected |
| H2a | H2a_1 | TE >> L | 0.007 | 0.011 | 0.674 | 0.500 | Rejected |
|  | H2a_2 | EC >> L | 0.021 | 0.029 | 0.715 | 0.475 | Rejected |
|  | H2a_3 | RP >> L | 0.015 | 0.024 | 0.610 | 0.542 | Rejected |
|  | H2a_4 | ER >> L | 0.021 | 0.028 | 0.734 | 0.464 | Rejected |
| H2b | H2b_1 | TE >> AT | 0.001 | 0.013 | 0.049 | 0.961 | Rejected |
|  | H2b_2 | EC >> AT | 0.087 | 0.038 | 2.272 | 0.000^***^ | Supported |
|  | H2b_3 | RP >> AT | 0.089 | 0.034 | 2.617 | 0.000^***^ | Supported |
|  | H2b_4 | ER >> AT | 0.154 | 0.048 | 3.221 | 0.000^***^ | Supported |
| H3a | H3a_1 | WW >> L | 0.073 | 0.032 | 2.292 | 0.022^*^ | Supported |
|  | H3a_2 | OC >> L | 0.082 | 0.032 | 2.562 | 0.015^*^ | Supported |
|  | H3a_3 | OV >> L | 0.083 | 0.036 | 2.313 | 0.021^*^ | Supported |
|  | H3a_4 | CS >> L | 0.069 | 0.029 | 2.373 | 0.021^*^ | Supported |
|  | H3a_5 | MC >> L | 0.074 | 0.030 | 2.467 | 0.017^*^ | Supported |
| H3b | H3b_1 | WW >> AT | 0.059 | 0.028 | 2.107 | 0.024^*^ | Supported |
|  | H3b_2 | OC >> AT | 0.084 | 0.034 | 2.471 | 0.018^*^ | Supported |
|  | H3b_3 | OV >> AT | 0.140 | 0.050 | 2.800 | 0.008^**^ | Supported |
|  | H3b_4 | CS >> AT | 0.118 | 0.028 | 4.214 | 0.000^***^ | Supported |
|  | H3b_5 | MC >> AT | 0.075 | 0.035 | 2.143 | 0.026^*^ | Supported |
| H4a | H3a_1 | LP >> L | 0.071 | 0.031 | 2.290 | 0.019^*^ | Supported |
|  | H3a_2 | UT >> L | 0.067 | 0.028 | 2.393 | 0.017^*^ | Supported |
|  | H3a_3 | WAO >> L | 0.083 | 0.020 | 4.150 | 0.000^***^ | Supported |
| H4b | H3b_1 | LP >> AT | 0.080 | 0.030 | 2.664 | 0.020^*^ | Supported |
|  | H3b_2 | UT >> AT | 0.062 | 0.028 | 2.214 | 0.039^*^ | Supported |
|  | H3b_3 | WAO >> AT | 0.074 | 0.029 | 2.552 | 0.023^*^ | Supported |
| H4c | H4c_1 | LP >> WW | 0.007 | 0.020 | 0.354 | 0.724 | Rejected |
|  | H4c_2 | UT >> WW | 0.023 | 0.019 | 1.251 | 0.211 | Rejected |
|  | H4c_3 | WAO >> WW | 0.084 | 0.041 | 2.064 | 0.039^*^ | Supported |
|  | H4c_4 | LP >> OC | 0.029 | 0.026 | 1.111 | 0.266 | Rejected |
|  | H4c_5 | UT >> OC | 0.054 | 0.025 | 2.047 | 0.041^*^ | Supported |
|  | H4c_6 | WAO >> OC | 0.078 | 0.038 | 2.040 | 0.040^*^ | Supported |
|  | H4c_7 | LP >> OV | 0.056 | 0.026 | 2.141 | 0.032^*^ | Supported |
|  | H4c_8 | UT >> OV | 0.017 | 0.014 | 1.192 | 0.233 | Rejected |
|  | H4c_9 | WAO >> OV | 0.070 | 0.031 | 2.213 | 0.027^*^ | Supported |
|  | H4c_10 | LP >> CS | 0.097 | 0.036 | 2.689 | 0.007^**^ | Supported |
|  | H4c_11 | UT >> CS | 0.027 | 0.021 | 1.274 | 0.203 | Rejected |
|  | H4c_12 | WAO >> CS | 0.024 | 0.029 | 0.828 | 0.408 | Rejected |
|  | H4c_13 | LP >> MC | 0.028 | 0.025 | 1.115 | 0.265 | Rejected |
|  | H4c_14 | UT >> MC | 0.018 | 0.019 | 0.986 | 0.324 | Rejected |
|  | H4c_15 | WAO >> MC | 0.029 | 0.027 | 1.065 | 0.287 | Rejected |
| H5 | | PU >> L | 0.086 | 0.040 | 2.087 | 0.039^*^ | Supported |
| H6 | | PEU >> L | 0.021 | 0.040 | 0.514 | 0.607 | Rejected |
| H7 | | AT >> L | 0.037 | 0.031 | 1.225 | 0.221 | Rejected |
| H8 | | PEU >> PU | 0.028 | 0.036 | 0.784 | 0.433 | Rejected |
| H9 | | PU >> AT | 0.092 | 0.042 | 2.187 | 0.034^*^ | Supported |
| H10 | | PEU >> AT | 0.021 | 0.024 | 0.863 | 0.388 | Rejected |
| H11 | | PU >> OSI | 0.001 | 0.024 | 0.021 | 0.983 | Rejected |
| H12 | | SN >> OSI | 0.017 | 0.031 | 0.546 | 0.585 | Rejected |
| H13 | | PBC >> OSI | 0.003 | 0.007 | 0.360 | 0.719 | Rejected |
| H14 | | AT >> OSI | 0.091 | 0.026 | 3.498 | 0.000^***^ | Supported |
| H15 | H15_1 | C >> OSI | 0.014 | 0.017 | 0.838 | 0.402 | Rejected |
|  | H15_2 | VC >> OSI | 0.271 | 0.076 | 3.557 | 0.000^***^ | Supported |
|  | H15_3 | IBB >> OSI | 0.132 | 0.046 | 2.839 | 0.005^**^ | Supported |
|  | H15_4 | SE >> OSI | 0.018 | 0.021 | 0.873 | 0.383 | Rejected |
|  | H15_5 | SBE >> OSI | 0.063 | 0.020 | 3.148 | 0.000^***^ | Supported |
| H16 | H16_1 | TA >> C | 0.005 | 0.033 | 0.150 | 0.881 | Rejected |
|  | H16_2 | TM >> C | 0.008 | 0.021 | 0.379 | 0.705 | Rejected |
|  | H16_3 | TA >> VC | 0.022 | 0.30 | 0.756 | 0.449 | Rejected |
|  | H16_4 | TM >> VC | 0.008 | 0.016 | 0.527 | 0.597 | Rejected |
|  | H16_5 | TA >> IBB | 0.007 | 0.006 | 1.167 | 0.267 | Rejected |
|  | H16_6 | TM >> IBB | 0.014 | 0.030 | 0.470 | 0.639 | Rejected |
|  | H16_7 | TA >> SE | 0.010 | 0.049 | 0.193 | 0.847 | Rejected |
|  | H16_8 | TM >> SE | 0.034 | 0.043 | 0.787 | 0.431 | Rejected |
|  | H16_9 | TA >> SBE | 0.004 | 0.056 | 0.072 | 0.943 | Rejected |
|  | H16_10 | TM >> SBE | 0.077 | 0.041 | 1.853 | 0.064 | Rejected |
| H17 | H17_1 | NDI >> C | 0.010 | 0.017 | 0.582 | 0.561 | Rejected |
|  | H17_2 | NDI >> VC | 0.008 | 0.015 | 0.575 | 0.565 | Rejected |
|  | H17_3 | NDI >> IBB | 0.008 | 0.025 | 0.323 | 0.747 | Rejected |
|  | H17_4 | NDI >> SE | 0.056 | 0.026 | 2.156 | 0.031^*^ | Supported |
|  | H17_5 | EF >> SBE | 0.069 | 0.025 | 2.761 | 0.002^**^ | Supported |
|  | H17_6 | OCO >> C | 0.001 | 0.002 | 0.748 | 0.454 | Rejected |
|  | H17_7 | OCO >> VC | 0.018 | 0.019 | 0.985 | 0.357 | Rejected |
|  | H17_8 | OCO >> IBB | 0.000 | 0.001 | 0.424 | 0.672 | Rejected |
|  | H17_9 | OCO >> SE | 0.000 | 0.001 | 0.018 | 0.986 | Rejected |
|  | H17_10 | OCO >> SBE | 0.000 | 0.001 | 0.430 | 0.667 | Rejected |
| H18 | H18_1 | SDF >> C | 0.048 | 0.017 | 2.823 | 0.001^***^ | Supported |
|  | H18_2 | SDF >> VC | 0.001 | 0.013 | 0.062 | 0.951 | Rejected |
|  | H18_3 | SDF >> IBB | 0.003 | 0.023 | 0.147 | 0.883 | Rejected |
|  | H18_4 | SDF >> SE | 0.179 | 0.054 | 3.315 | 0.001^***^ | Supported |
|  | H18_5 | SDF >> SBE | 0.003 | 0.022 | 0.121 | 0.904 | Rejected |
| H19 | | OSI >> OSB | 0.069 | 0.031 | 2.226 | 0.031^*^ | Supported |

Note: ^*^α = 0.05; ^**^α = 0.01; ^***^α = 0.001. Source: own processing


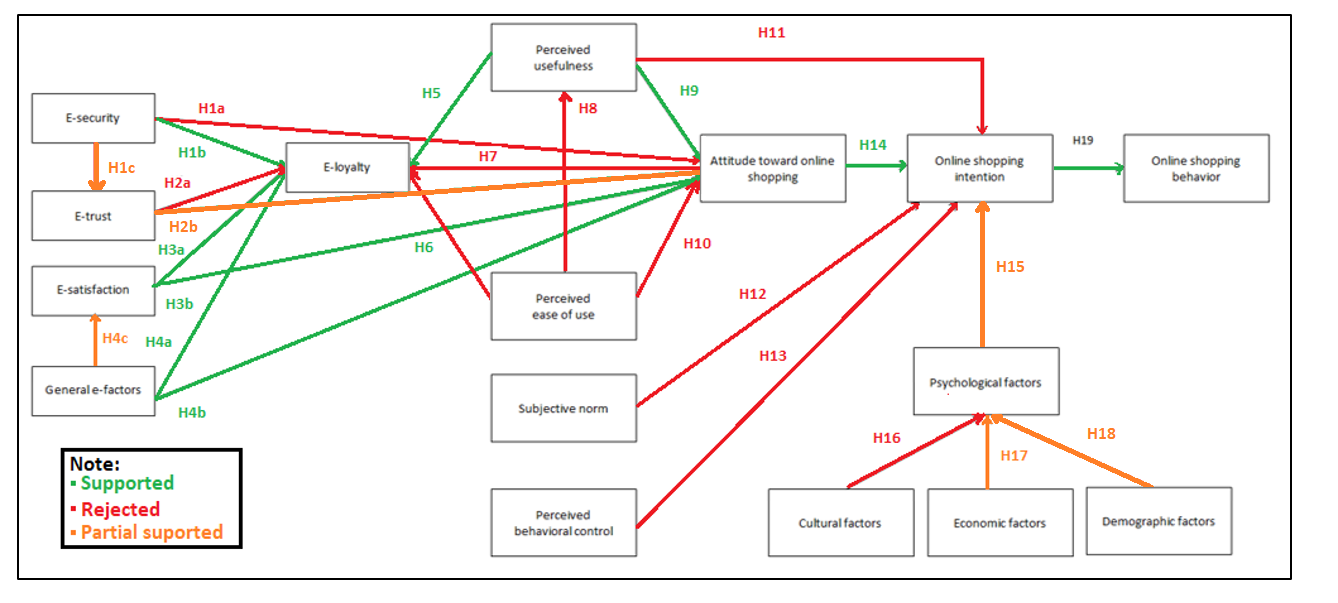


**S4 Figure 4. Evaluation of a comprehensive model of online shopping behavior** (Source: own processing)
