# Supplementary figures and images for: Modelling of online shopping behavior in the Czech online environment
Source: PLoS One. 2025 Jan 15;20(1):e0308725. doi: 10.1371/journal.pone.0308725 (PMC11734907; doi:10.1371/journal.pone.0308725)

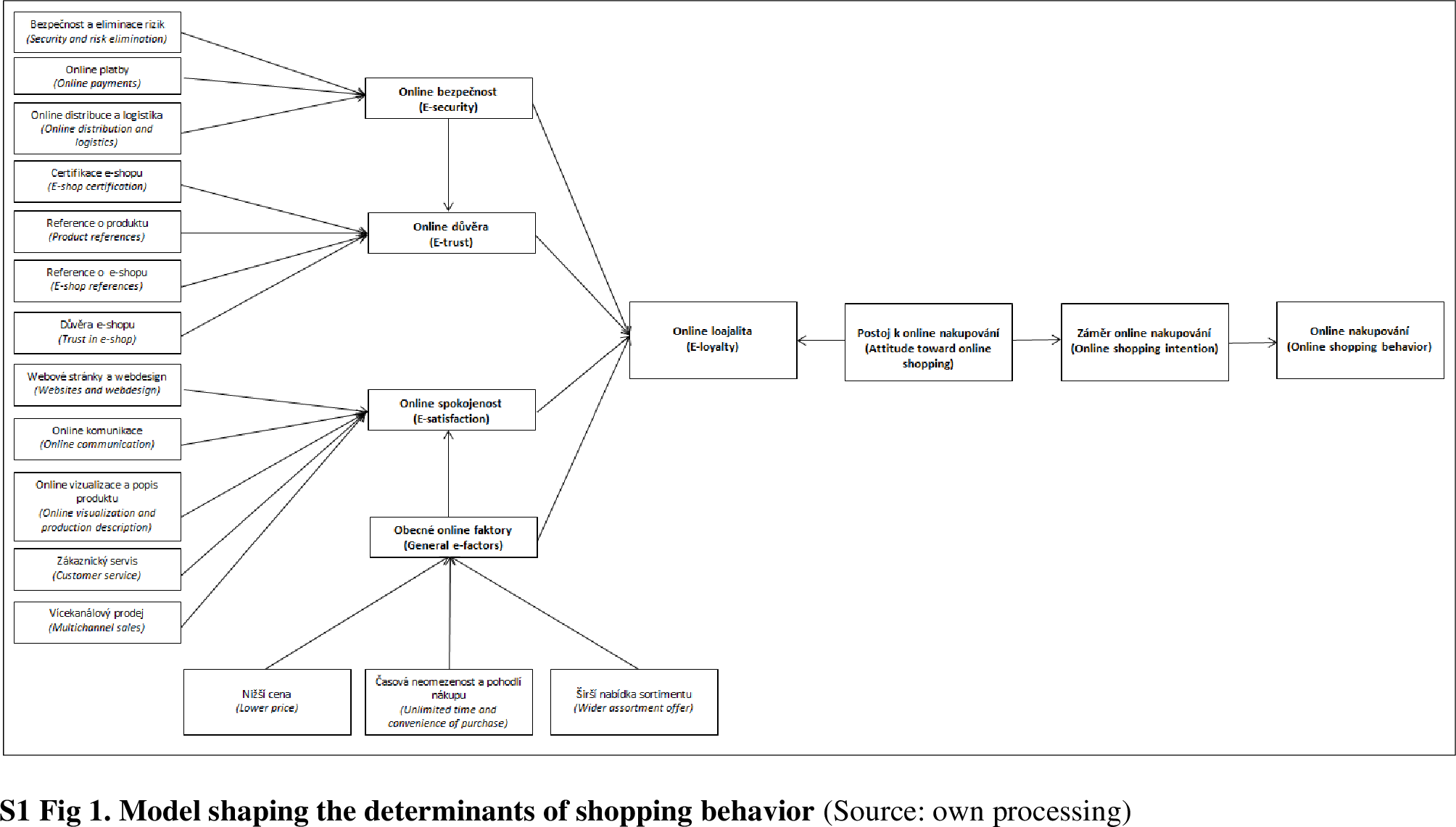

Supplement: S1 Fig — (TIF) [file pone.0308725.s002.tif]

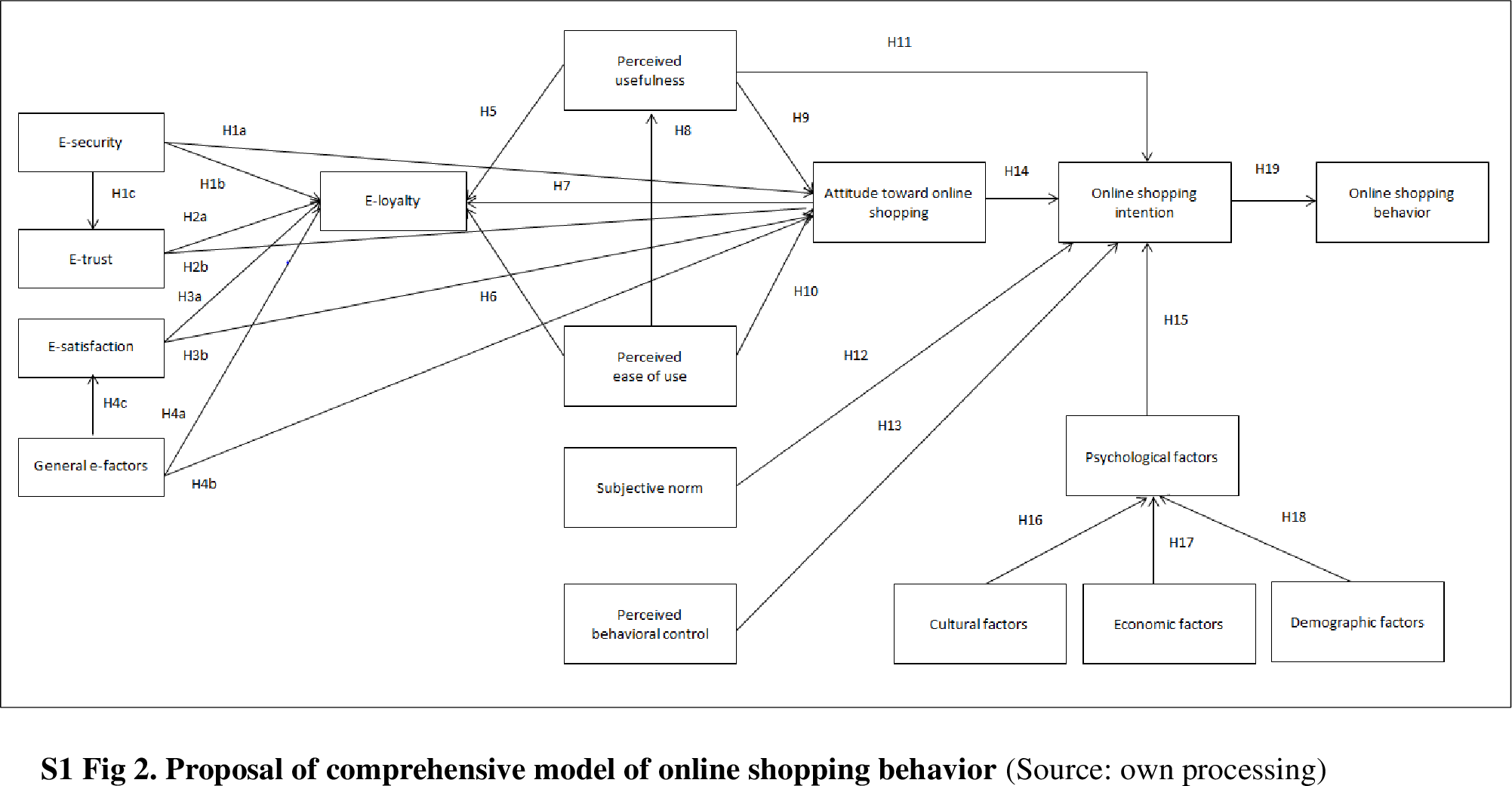

Supplement: S2 Fig — (TIF) [file pone.0308725.s003.tif]

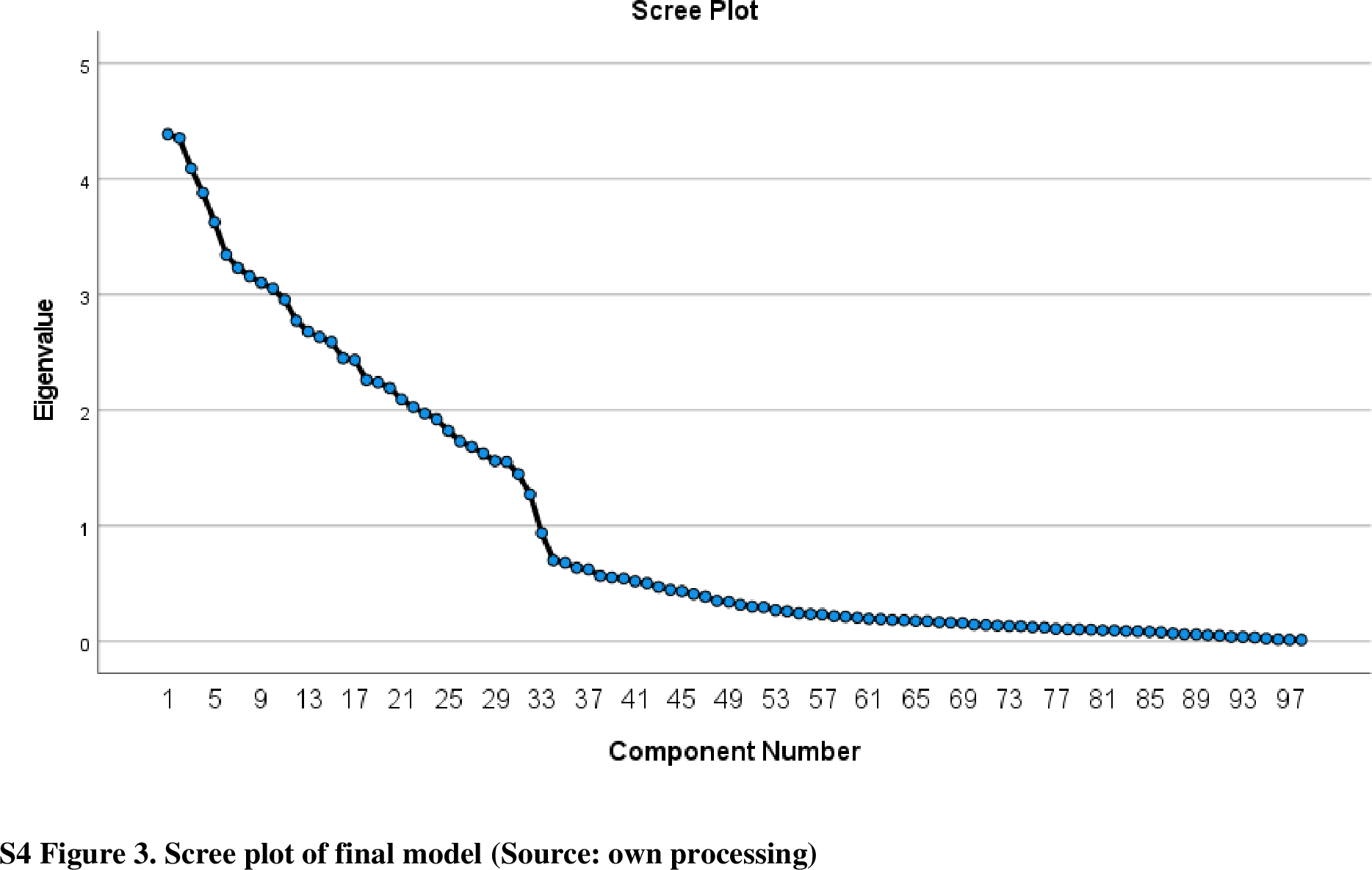

Supplement: S3 Fig — (TIF) [file pone.0308725.s004.tif]

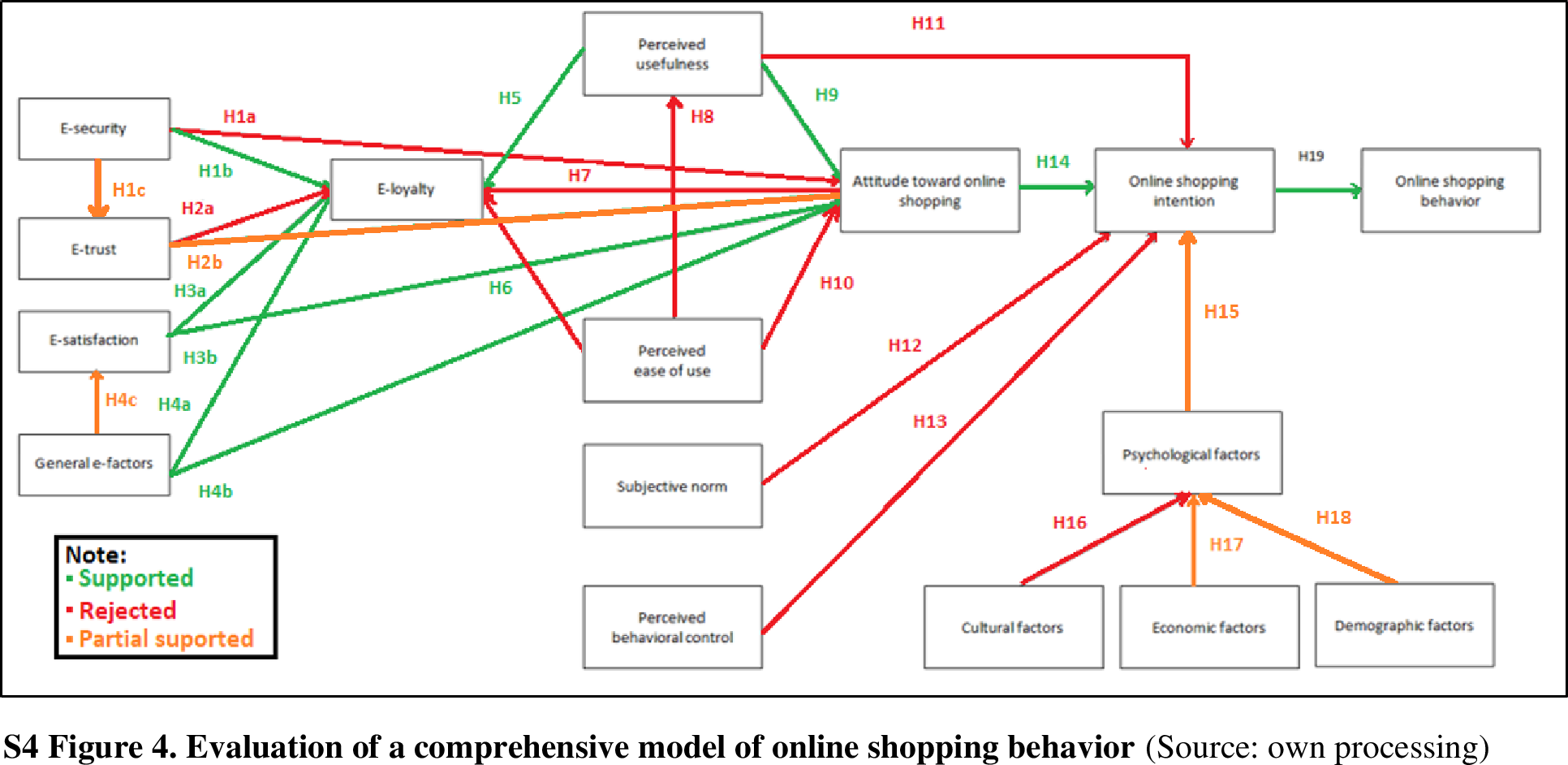

Supplement: S4 Fig — (TIF) [file pone.0308725.s005.tif]
